# Factors associated with diversity, quantity and zoonotic potential of ectoparasites on urban mice and voles

**DOI:** 10.1371/journal.pone.0199385

**Published:** 2018-06-25

**Authors:** Denny Maaz, Jürgen Krücken, Julia Blümke, Dania Richter, Janina McKay-Demeler, Franz-Rainer Matuschka, Susanne Hartmann, Georg von Samson-Himmelstjerna

**Affiliations:** 1 Institute for Parasitology and Tropical Veterinary Medicine, Freie Universität Berlin, Berlin, Germany; 2 Institute of Immunology, Freie Universität Berlin, Berlin, Germany; 3 Institute of Geoecology, Technische Universität Braunschweig, Germany; 4 Outpatient Clinic, University of Potsdam, Potsdam, Germany; University of Pretoria, SOUTH AFRICA

## Abstract

Wild rodents are important hosts for tick larvae but co-infestations with other mites and insects are largely neglected. Small rodents were trapped at four study sites in Berlin, Germany, to quantify their ectoparasite diversity. Host-specific, spatial and temporal occurrence of ectoparasites was determined to assess their influence on direct and indirect zoonotic risk due to mice and voles in an urban agglomeration. Rodent-associated arthropods were diverse, including 63 species observed on six host species with an overall prevalence of 99%. The tick *Ixodes ricinus* was the most prevalent species, found on 56% of the rodents. The trapping location clearly affected the presence of different rodent species and, therefore, the occurrence of particular host-specific parasites. In Berlin, fewer temporary and periodic parasite species as well as non-parasitic species (fleas, chiggers and nidicolous Gamasina) were detected than reported from rural areas. In addition, abundance of parasites with low host-specificity (ticks, fleas and chiggers) apparently decreased with increasing landscape fragmentation associated with a gradient of urbanisation. In contrast, stationary ectoparasites, closely adapted to the rodent host, such as the fur mites Myobiidae and Listrophoridae, were most abundant at the two urban sites. A direct zoonotic risk of infection for people may only be posed by *Nosopsyllus fasciatus* fleas, which were prevalent even in the city centre. More importantly, peridomestic rodents clearly supported the life cycle of ticks in the city as hosts for their subadult stages. In addition to trapping location, season, host species, body condition and host sex, infestation with fleas, gamasid Laelapidae mites and prostigmatic Myobiidae mites were associated with significantly altered abundance of *I*. *ricinus* larvae on mice and voles. Whether this is caused by predation, grooming behaviour or interaction with the host immune system is unclear. The present study constitutes a basis to identify interactions and vector function of rodent-associated arthropods and their potential impact on zoonotic diseases.

## Introduction

Commensal rodents, such as house mice (*Mus musculus*) or Norway or black rats (*Rattus norvegicus* and *Rattus rattus*), living inside buildings, are often considered to be the principal risks of zoonotic infections for humans. However, bank voles (*Myodes glareolus*) and *Apodemus* mice are also known to enter basements and storage areas during winter [1–3]. These and other mice and voles live in close proximity to humans and are abundant in parks and other greenspaces of urban agglomerations. The population density of these “peridomestic” rodents, such as the striped field mouse, *Apodemus agrarius*, appears to be even higher in urban than rural regions, due to a prolonged breeding season and better winter survival [4]. Changes in the seasonality of the rodent hosts in terms of reproduction, abundance, behaviour and motility may affect the seasonal abundance of rodent-associated arthropods. Also, behavioural differences of the host, such as changes in circadian rhythm and home range as well as an increased longevity, were observed in urban areas [4]. This may also affect the species diversity and quantity of rodent-associated arthropods. In the last decades, the geographical distribution of arthropod vectors changed [5, 6]. Monitoring of ectoparasite communities of wild animals may provide important information on such processes. Since the proportion of people living in urban areas is constantly increasing worldwide, this is especially important in human agglomerations.

Peridomestic rodents spread and maintain the enzootic cycles of tick-borne pathogens in cities [7–9]. Although Lyme-*Borrelia*, spotted-fever *Rickettsia* spp., tick-borne encephalitis virus and other pathogens have been detected in rodent ectoparasites other than ticks [10–13], their vector competence has not been verified. Assessing the diversity and quantity of other mite and insect species on rodent hosts might provide a basis for studies on vector competence of the most abundant arthropod species to elucidate their role in enzootic cycles. Furthermore, the examination of different ectoparasite groups co-occurring with ticks allows to evaluate their effects on tick infestation.

The aim of the present study was (I) to determine and quantify the total species diversity of arthropods located in the fur and on the skin of peridomestic rodents in Berlin, Germany, (II) to examine their distribution in respect to host species, trapping location/urbanisation, and seasonality, (III) to assess the zoonotic risk due to these arthropods for people in urban agglomerations and (IV) to determine parameters affecting the abundance of *Ixodes ricinus* larvae on peridomestic mice and voles.

## Materials and methods

### Ethics statement

Rodent trapping and euthanasia were performed in accordance with the German laws on animal protection (*Tierschutzgesetz*) and nature conservation (*Bundesnaturschutzgesetz*) and were approved by the *Landesamt für Gesundheit und Soziales* (*LAGeSo*) Berlin under the registration number G 0256/10 and the *Obere Naturschutzbehörde* Berlin under the reference number I E 210(V)–OA-SG/LSG2a/602;OA-AS/G/825. The three mouse species from the genus *Apodemus* included “besonders geschützte Arten” (= specially protected species) according to the German *Bundesnaturschutzgesetz*.

### Rodent trapping

**Fig 1:**
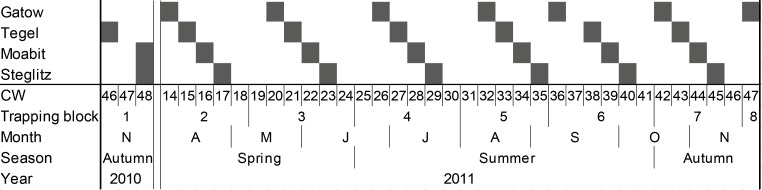
Trapping schedule. Trapping blocks and time as calendar week (CW), month, season and year (columns) for every trapping location (rows). The season was categorised by means of trapping blocks. Trapping occasions with three consecutive trap nights are shown in grey.

Wild rodents were trapped at four study sites in Berlin in November 2010 and between April and November 2011. Two forest sites at the periphery were chosen as periurban sites with limited human influence (Gatow and Tegel), while two urban study sites were situated in the densely populated city (Steglitz and Moabit). The trapping location Gatow (N52° 28.167 E013° 08.460) is a wooded area at the General-Steinhoff-Barracks characterised by pinewood with a few oaks and sparse ground vegetation. Traps were placed along paths and in an adjacent meadow situated at the margin of the former runways. Another study site was situated within a large forested area in the district Tegel (N52° 36.351 E013° 16.288). Trapping was performed in the surroundings of the forestry office and along a wide path through the forest. The forest comprised mainly pines, beeches, oaks and little ground vegetation. The Botanic Garden Berlin in the district Steglitz comprises about 43 ha and is surrounded by widely spaced single-family houses with garden properties (N52° 27.233 E013° 18.151). The park is tended by gardeners and watered during dry periods. The vegetation of this study site is characterised by widely-spaced old trees, many shrubs and a dense ground layer of ivy and ground elder [14]. The most urban study site in the district Moabit was a backyard of an apartment building in the densely populated city centre (N52° 31.286 E013° 21.571). Trapping was performed in six-week blocks, with one site visited per week (termed “occasions” henceforth), followed by two weeks without trapping (Fig 1). In total, every site was sampled during seven occasions. Rodents were trapped for three consecutive nights at each occasion. Traps were emptied and cleaned in the morning and deactivated during the daytime. Before trapping, they were filled with cotton, rodent pellets and a piece of apple.

Initially, 30 Longworth live traps and 10 Tomahawk rat live traps were placed at every location. According to the size of the location, the number of Longworth traps was increased to 40 traps in Steglitz and to 50 traps in Gatow and Tegel in July 2011. Because only two persons were available for the necropsies, no more than eight rodents were examined after each trap night. Therefore, the traps were checked according to random matrices and only the first eight animals were examined and the remaining rodents were released.

Trapped rodents were anaesthetised intraperitoneally with 0.1 mg/g ketamine and 0.012 mg/g xylazine and subsequently euthanised by cervical dislocation followed by cardiac bleeding into serum tubes. The rodents were wrapped in individual plastic bags and traps as well as the cotton wool were thoroughly screened for fleas, gamasid mites and other detached arthropods. Animals were placed on heat packs (approximately 38°C) to improve the survival of the parasites and transferred to the Institute for Parasitology and Tropical Veterinary Medicine for necropsies. After each week of trapping, the traps were cleaned, cotton wool and food were removed.

### Necropsies

In the laboratory, animals were placed on electric heating blocks at 38°C and species, sex, body weight and size were determined. For some animals (especially *Microtus*), skulls were prepared and teeth morphology was used for species determination [15].

The fur and skin of the animals were thoroughly screened for arthropods under a stereo microscope with clean forceps from head to tail. Approximately 15 minutes were spent per animal. In addition, the plastic bags used for transportation were checked. Whereas ticks attached to the skin remained there, detached ticks were transferred into glass vials with screened caps and placed in a desiccator filled with oversaturated magnesium sulphate solution to allow moulting to the next life stage. All other arthropods were preserved in tubes with 70% ethanol, except for rodents heavily infested with Myocoptidae or Listrophoridae, of which not all specimens were sampled. During a necropsy, additional organ samples were obtained for other studies and the reproduction status of female rodents was determined by checking for embryos. After removal of the eyes for age determination (described in Krücken et al. [16]), the remaining carcasses were placed in glass beakers over water to allow detachment of still attached ticks and to improve the recovery of small arthropods. The water and the rodent bodies were examined microscopically during the following week and remaining ticks were placed in the desiccator, whereas other arthropods were preserved in 70% ethanol. After six weeks, any ticks from the desiccator were transferred to 70% ethanol.

### Arthropod species determination

The preserved arthropods were determined to species level wherever possible with the help of a stereo microscope and a microscope with up to 1000× magnification. Several specimens of fleas and gamasid mites were cleared in 10% potassium hydroxide before examination. Different literature was used for species determination of fleas [17–20], lice [21], ticks [22–26], Gamasina (Mesostigmata) [27–30], Myobiidae [31–33], Trombiculidae [34–36], Cheyletidae [37, 38], Pygmephoridae [39, 40], Ereynetidae [41, 42], Myocoptidae [43–47], Listrophoridae [48–51], Gastronyssidae [52, 53], Glycyphagidae and Acaridae [54–58]. For rodents, on which nits of sucking lice containing nymphs were observed but no postembryonal life stages, the number of lice for measures of prevalence and mean intensity was set to one for the respective species. Similarly, for closed eggs of Myobiidae and Myocoptidae despite absence of postembryonal stages, specimen number was set to one for the respective family.

### Seasonality

To compare the seasonal occurrence between the different rodent-associated arthropods, abundance was normalised to host species and location. This avoided that strictly host-specific parasites appear to be more abundant in seasons when their preferred host was trapped more frequently. In order to analyse representative data, only the following four rodent subsets (species-location-combinations) were included for which two or more animals were caught in at least five out of six trapping occasions: *Myodes glareolus* in Gatow, *Apodemus flavicollis* in Gatow, *A*. *flavicollis* in Steglitz und *Apodemus agrarius* in Steglitz. Abundance of the four subsets were averaged, 152 trapped rodents from six trapping blocks (at least eight rodents each) from April to November 2011 were used for the analysis. The “normalised mean abundance” was calculated as the mean of the mean abundance of every rodent subset to prevent overweighing one factor. The standard error of the mean was calculated, whereby the respective mean abundance of every subset was subtracted from the parasite counts to generate the sums of deviation squares. Only those parasite species were included in the analysis, which reached an overall prevalence of at least 10%, i.e. they were detected on at least 15 animals. Hence, the normalised mean abundance for every arthropod was adjusted for better visualisation of the time course of occurrence and the y axes are not shown because the interpretation of the normalised values would be unreasonable.

### Statistics

As the mean abundance (mean number of parasites on all screened rodents) provides only limited information about the parasite distribution across host species and other parameters, mainly prevalence (number of infested rodents divided by total number of screened rodents) and mean intensity (mean number of parasites on infested rodents) were used in combination, as:

prevalence×meanintensity=meanabundance.

Local differences in prevalence and intensity of rodent-associated arthropods often depended on the presence of certain rodent host species, particularly for host-specific parasites. Therefore, regression analyses served to identify associations with either host species (n = 6) or host family (voles/mice) and trapping location (n = 4) or location category (urban/periurban). In order to model prevalence data, logistic regression analyses (LRA) were calculated with the parasite occurrence (infested/non-infested) as dependent variable and trapping location (or location category: urban/periurban) and host species (or rodent family: voles/mice) as independent variables. For correction of under- or overdispersion, LRAs were then recalculated with their respective dispersion parameter σ^2^ instead of 1, which was calculated from the sum of squares of the residuals divided by the degrees of freedom. For modelling mean infestation intensities, a negative binomial regression analysis (NRA) was used with the ectoparasite counts of infested hosts as dependent variable and trapping location and host species as independent variables. Non-infested animals were excluded from the NRA. The negative binomial distribution appears to be the most adequate approach for the analysis of distribution of most ectoparasitic groups [59, 60]. The combination of both regression methods in a zero-inflated negative binomial regression analysis of the abundance was not successful, presumably because of missing data for several host species-location-combinations, collinearity between both factors and small sample size. For both regression analysis methods, the odds ratio (OR) and rate ratio (RR) of the level of interest with the corresponding p-value of the F statistics are presented in the text and the full model with all odds ratios and rate ratios with 95% confidence intervals (95% CI), p-values and model dispersion parameters σ^2^ (LRA) or θ (NRA) are presented in S1 Table.

For statistical testing of differences between groups of rodents within one trapping location or host species, Mann-Whitney-U-test was used for the nonparametric mean intensities (two groups) and mid-p-exact-test for prevalence, with Holm-corrected post-tests if more than 2 groups were analysed.

For the analysis of parameters affecting the number of *I*. *ricinus* larvae on wild rodents, a generalised linear mixed model (GLMM) with negative binomial distribution was performed including all screened rodents with the exception of *Microtus agrestis* voles (n = 2). The infestation parameters prevalence and intensity were combined to abundance and not separately analysed to increase statistical power. Modelling was limited to the larval stage because nymphs were only found on every 6^th^ rodent. After exclusion of rodents with missing values (including all from 2010), 219 rodents trapped in 2011 were used for the analysis. The counts of *I*. *ricinus* larvae on the rodents were used as dependent variable. The interaction of trapping location (4 levels) and season (3 levels) was treated as random effect, while host species (5 levels), host characteristics (sex, body condition, age) and co-infestations with other frequent ectoparasites (counts of fleas, lice, parasitic laelapid mites, myobiid mites, trombiculid mites, myocoptid mites and listrophorid mites) were included as fixed effects. The season was categorised (see Fig 1) into “spring” (calendar week (CW) 14–24 including 2 trapping blocks, n = 33), “summer” (CW 25–41 including three trapping blocks, n = 125) and “autumn” (CW 42–47 with 2 trapping occasions in Gatow and one at the other locations, n = 61). Since explanatory variables must be statistically independent and many host characteristics are correlated with each other, three condensed parameters as used in Rossin et al. [61] were included: (I) The sex, including reproductive condition, was used in the three levels male (n = 115), non-pregnant female (n = 64) and pregnant female (n = 40). (II) The different methods for age estimation (e.g. head-body-length, weight) most strongly depend on the season of birth and sexual dimorphism and are thus inappropriate in mature animals. The strongest, nearly linear relationship was described between the age and the weight of the formol-fixed and dried eye lenses at least for *Apodemus sylvaticus* [62], *A*. *agrarius* [63] and *Microtus arvalis* [64]. Since calibration curves were not available for all trapped rodent species, z-transformations of the mean weight of both lenses for every rodent species were used as a proxy for age to ensure independence of the variable from host species. To calculate z-values, the weights of the dried eye lenses subtracted by the mean weight of the dried eye lenses of the same species was divided by the standard deviation of the latter. (III) In order to estimate the nutritional or body condition, an adaptation of the method in Rossin et al. [61] was used, where the differences (residuals) from an average weight to body-length ratio were calculated using linear regression. A regression of the log of the body weight against the head-body-length was calculated. However, as voles and mice differ morphologically and residuals of voles and mice of a combined linear regression differ significantly (unpaired t test: p<0.001), but not within species of each group (mice: one-way ANOVA of three *Apodemus* spp.: p = 0.09, voles: unpaired t test of *M*. *glareolus* and *M*. *arvalis*: p = 0.25), separate linear regressions were used for either (mice: log(weight in g) = 0.3964 + 0.0285 × head-body-length in mm, adjusted R^2^ = 0.83, p<0.001; voles: log(weight in g) = 0.3696 + 0.0277 × head-body-length in mm, adjusted R^2^ = 0.66, p<0.001). Positive residuals from the calculated curves indicate a good body condition, because rodents are comparatively heavier than expected for their body length; negative residuals indicate a low nutritional status.

The GLMM regression analysis started with the full model including all variables followed by backward variable selection. In the process, variables were successively excluded which revealed the highest p-value until all remaining variables were significant.

For the calculation of the 95% CI of mean intensities, a bootstrapping method was performed with 2,000 bootstrap replications calculating bias-corrected and accelerated confidence intervals in the online software Quantitative Parasitology 3.0 [65]. Wilson-Score confidence intervals from proportions were used for prevalence. Wilson-Score confidence intervals (package PropCIs), mid-P-exact-test (package epitools), χ^2^-test, one-way and two-way ANOVA, generalised linear models for logistic regression analysis and negative binomial regression analysis (package MASS), negative binomial GLMM (package lme4) and calculation of variance inflation factors (package faraway) were performed in R Statistics. D’Agostini-Pearson omnibus test for normality was recommended and performed in GraphPad Prism 7.

## Results

Other data derived from this rodent trapping campaign have been published previously [16, 66, 67].

### Rodent sampling

During November 2010 and from April to November 2011, 276 mice and voles belonging to six species were trapped at four study sites in Berlin. Of those, 256 were thoroughly screened for ectoparasites, whereas nineteen animals were released (18 in Steglitz, one in Gatow) and one fell victim to carnivores (in Steglitz). During the seven trapping occasions at each of the sites, the highest trapping success with 122 rodents was achieved at the urban Steglitz site, followed by the periurban forest habitat in Gatow (Table 1). The forest site Tegel and the most urbanized site, Moabit in Central Berlin, exhibited the lowest rodent trapping success with only 36 and 26 rodents, respectively. The highest activity of rodents was observed in June and July 2011 with a second peak for some species in October/November (S1 Fig).

**Table 1:** Trapped rodents at the study sites.

Trapping location	Rodent species						Total
	*Myodes*	*Microtus*		*Apodemus*			
	*glareolus*	*agrestis*	*arvalis*	*agrarius*	*flavicollis*	*sylvaticus*	
Moabit	1	-	-	-	-	25	26
Steglitz	-	-	4	88	30	-	122
Tegel	14	-	-	-	22	-	36
Gatow	44	2	7	5	34	-	92
Total	59	2	11	93	86	25	276

Number of mice and voles for every trapped species (columns) is depicted for the four study sites (rows). Total specimen number of each rodent species and of all rodent species at each trapping location are shown in the last row or column, respectively. The hyphen indicates absence of the respective rodent.

The wood mouse *A*. *sylvaticus* was exclusively found in Moabit and constituted the dominant rodent species at this site. The two sylvatic species, yellow-necked mouse (*A*. *flavicollis*) and bank vole (*M*. *glareolus*), were the most frequent species in Gatow and Tegel, although *A*. *flavicollis* was also regularly trapped in Steglitz. Nevertheless, the striped field mouse (*A*. *agrarius*) was the dominant species at this study site. At the only other site where *A*. *agrarius* was trapped, in Gatow, it was captured in low numbers. The field vole (*M*. *agrestis*) and the common vole (*M*. *arvalis*) were rarely caught in Gatow and Steglitz. The occurrence of a single *M*. *glareolus* in Moabit seems to be an exception.

### The diversity of rodent-associated arthropods

**Fig 2:**
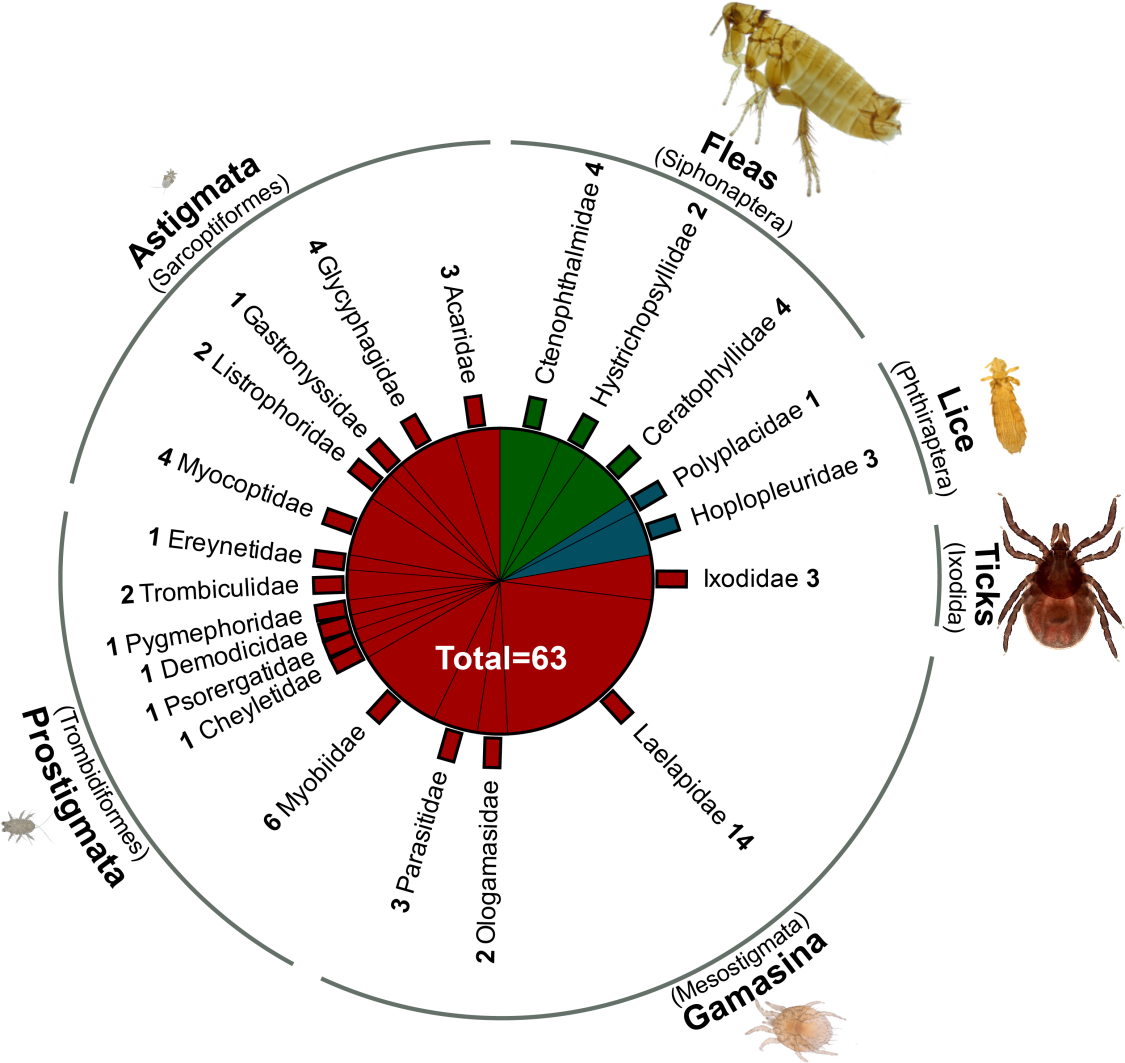
Taxonomic distribution of 63 detected species across families and higher taxa of arthropods. Numbers of species are accompanied by family names. Families shown in red belong to mites (Acari), those in blue to lice (Phthiraptera) and those in green to fleas (Siphonaptera). Parasite micrographs show representative specimens from the different groups depicted in the same size ratio.

From 256 examined mice and voles, a total of 5,429 arthropods belonging to 63 different species was collected from fur and skin and identified (Fig 2 and S2 Table). Taxonomic arthropod groups of different levels (order/suborder/family) were compared and grouped according to phylogenetic position and feeding habits. The arthropods represented two orders of Insecta, fleas (Siphonaptera, ten species) and lice (Phthiraptera, four species, all suborder Anoplura), but most importantly four orders of mites (Acari, 49 species). The latter were comprised of three species of hard ticks (Ixodida), 19 species of Mesostigmata (all cohort Gamasina), 13 species of Trombidiformes (all suborder Prostigmata) and 14 species of Sarcoptiformes (all cohort Astigmata). These species ranged in body sizes from 0.2 (astigmate mite: *Trichoecius tenax*) to 8 mm (flea: *Hystrichopsylla orientalis*).

The diversity of rodent-associated arthropods consisted of facultative and obligatory ectoparasites (fleas, lice and mites, 49 species) and also included phoretic life stages of non-parasitic arthropods (four mite species) and nidicolous mites, rarely occurring in the fur of rodents (10 mite species). The phoretic mites comprised life stages attached to the hair of rodents, such as female *Pygmephorus forcipatus* and deutonymphal hypopi of *Xenoryctes krameri* and *Glycyphagus hypudaei* (S2 Fig), as well as the hypopial stages of *Acarus nidicolous*, which were found phoretic on fleas (S2 Fig). Parasitic beetles from the family Leptinidae (*Leptinus testaceus*) and myasis-causing flies from the family Oestridae (*Oestromyia* spp.) found in other areas of Germany [68–70] were absent on rodents trapped in the present study.

**Fig 3:**
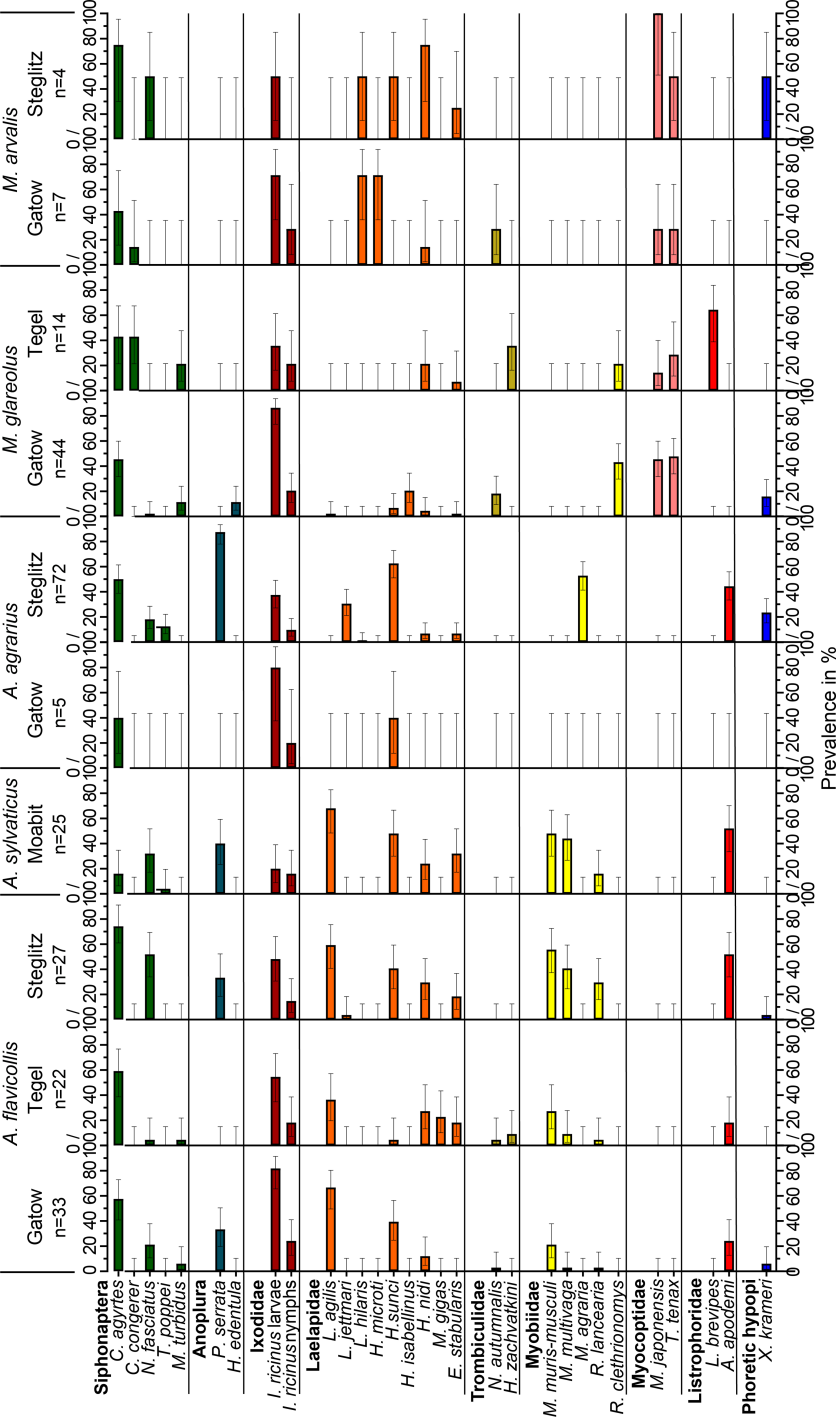
Prevalence of the most frequently observed rodent-associated arthropods. Bar plots with 95% CI showing percentage of infested rodent for every arthropod species on five rodent host species trapped at four trapping locations. One *M*. *glareolus* (Moabit) and two *M*. *agrestis* voles (Gatow) are not shown because of the small sample size. Only arthropod species are depicted which occurred at least five times on one of the illustrated host-location-combinations. Numbers below rodent species names depict the sample size (n) of examined mice or voles for every study site. Horizontal solid lines border species of the same parasite group.

Arthropods, in general, and ectoparasites, in particular, infested virtually every mouse and vole and amounted to an overall prevalence of 99% with an average of 16 specimens per host. The most frequent ectoparasite groups were Laelapidae (67%, Gamasina), fleas (63%), hard ticks (57%, Ixodida), Myobiidae (47%, Prostigmata), sucking lice (41%, Anoplura), Listrophoridae (32%, Astigmata), Myocoptidae (20%, Astigmata) and Trombiculidae (7%, Prostigmata) (Table 2 and Fig 3). The skin-inhabiting mites *Demodex* sp. (Demodicidae, Prostigmata), *Psorergates* sp. (Psorergatidae, Prostigmata), *Lophioglyphus liciosus* (Glycyphagidae, Astigmata) and mites living in the nasal cavities (Gastronyssidae, Astigmata and Ereynetidae, Prostigmata) were detected as incidental findings during microscopical examination of scabbed skin and after placing the carcass of the examined rodents over water for one week, which allowed the mites to leave the body. To the best knowledge of the authors, the finding of the eight mite species *Laelaps jettmari*, *Hirstionyssus (Echinonyssus) sunci*, *Radfordia clethrionomys*, *Paraspeleognathopsis bakeri*, *Trichoecius widawaensis*, *Listrophorus brevipes*, *Yunkeracarus apodemi* and *Acarus nidicolous* represent first records for the German fauna.

**Table 2:** Distribution of ectoparasitic arthropod groups on wild rodents.

Ectoparasite				Rodent host species																												
Taxon	n	Sex ratio		*M*. *glareolus*				*M*. *arvalis*				*M*. *agrestis*				*A*. *agrarius*				*A*. *flavicollis*				*A*. *sylvaticus*				All Species				
				29/29 = 59^a^				4/7 = 11				0/2 = 2				44/33 = 77				41/41 = 82				14/11 = 25				132/123 = 256^a^				
				No	P [%]	95% CI	mI [n]	No	P [%]	95% CI	mI [n]	No	P [%]	95% CI	mI [n]	No	P [%]	95% CI	mI [n]	No	P [%]	95% CI	mI [n]	No	P [%]	95% CI	mI [n]	No	P [%]	95% CI	mI [n]	max
Siphonaptera (Fleas)	504	1:	1.2	41	70	57–80	2.0	8	73	43–90	3.1	1	50	9–90	2.0	46	60	49–70	3.0	56	68	58–77	4.1	9	36	20–55	2.7	161	62.9	56.8–68.6	3.1	15
Anoplura (Sucking lice)	463	1:	2.2	6	10	5–20	2.7	3	27	8–57	5.0	-		0–66		66	86	76–92	5.4	20	24	16–35	2.9	10	40	23–59	1.9	105	41.0	35.2–47.1	4.4	44
Ixodidae (Hard ticks)	1370	n.d.		44	75	62–84	8.1	8	73	43–90	27.0	2	100	34–100	10.5	31	40	30–51	5.4	54	66	55–75	11.0	6	24	11–43	2.5	145	56.6	50.5–62.6	9.5	108
Parasitic Laelapidae	987	1:	12.3	18	31	20–43	2.1	9	82	52–95	13.4	-		0–66		59	77	66–85	2.8	63	77	67–85	7.9	22	88	70–96	7.3	171	66.8	60.8–72.3	5.8	91
Myobiidae	444	1:	4.1	22	37	26–50	4.0	4	36	15–65	2.2	2	100	34–100	11.0	38	49	38–60	2.6	38	46	36–57	3.8	17	68	48–83	4.8	121	47.3	41.2–53.4	3.7	20
Trombiculidae	88	n.d.		13	22	13–34	4.8	2	18	5–48	3.0	1	50	9–90	1.0	-		0–5		3	4	1–10	6.0	-		0–13		19	7.4	4.8–11.3	4.6	38
Myocoptidae^b^	302	1:	3.5	35	59	47–71	≥6.8	7	64	35–85	≥7.7	1	50	9–90	≥2.0	6	8	4–16	≥1.0	1	1	0.2–7	≥1.0	-		0–13		50	19.5	15.1–24.8	≥6	≥29
Listrophoridae^b^	1057	n.d.		9	15	8–26	≥16.0	-		0–26		2	100	34–100	≥13.0	32	42	31–53	≥11.0	26	32	29–50	≥14.0	13	52	33–70	≥14.0	82	32.0	26.6–38.0	≥12.9	≥146

Total number and sex ratio (male: female) of parasites as well as number of infested rodents. For each parasite group, prevalence and mean intensity for six rodent species are shown. The number of examined rodents and the number of male/female are given below the species name. The last column shows values for the sum of all rodents. Hyphens represent absence of parasites. n: Number of parasites. No: Number of infested rodents. P [%]: Prevalence in %. 95% CI: 95% confidence interval. mI [n]: mean intensity = mean number of parasites on infested rodents. max: maximum of parasite intensities. n.d.: no adults observed or not determined.

^a^ Sex of one bank vole was not determined

^b^ Because of small body size of Myocoptidae and Listrophoridae, not all specimens were sampled when high intensities occurred. Values should be treated as minimum numbers. Sex ratio of Listrophoridae was not determined

### Infestation differences between host species and between trapping locations

Hematophagous fleas, of which only the parasitic adults were observed, occurred on four of the five regularly trapped rodent species at a comparable prevalence of 60–73% (Table 2). Only the wood mouse seemed to be less frequently infested (36%), although this may also result from an overall reduced flea density in Moabit. Mice carried slightly more fleas than voles (NRA vole-RR = 0.57, p<0.001, S1J Table). Especially the largest species, the yellow-necked mouse, hosted an average of 4.1 fleas per infested animal (Table 2). Most flea species revealed little host-specificity and the dominant species *Ctenophthalmus agyrtes* (S2 Fig), which represented nearly one third of all observed fleas, was found on all rodent species and also at every trapping location (Fig 3 and S2 Table). Another frequently observed flea *Nosopsyllus fasciatus* appeared to infest *Apodemus* species more frequently than voles (LRA: voles-OR = 0.27, p = 0.047, S1A Table). Whereas *Typhloceras poppei* solely occurred on *Apodemus* mice (Fig 3 and S2 Table), *Ctenophthalmus assimilis*, *Ctenophthalmus congerer* and *Peromyscopsylla sylvatica* only infested voles (S2 Table). Occurrence differed also at particular study sites. For instance, *C*. *assimilis*, *P*. *sylvatica* and *H*. *orientalis* were only detected in Gatow although their main host species were also trapped at other locations.

The stationary parasitic, hematophagous lice parasitized every rodent species, with the exception of the two field voles. They were found in a markedly host-specific manner, i.e. each parasite species was identified on only a single host genus (S2 Table). Prevalence was higher in mice than in voles (LRA: vole-OR = 0.25, p = 0.001, S1B Table), which was most obvious in comparison to bank voles (Table 2). The louse species *Polyplax serrata* (S2 Fig), accounting for 80% of all louse specimens, infested exclusively *Apodemus* mice with prevalence rates of up to 82% (*A*. *agrarius*). The spatial distribution of the sucking lice was surprisingly inconsistent. In fact, *Hoplopleura affinis* was the dominant louse species on the striped field mouse in Gatow, but absent on such hosts in Steglitz (mid-P-exact-test: p<0.001, S2 Table). In contrast, 88% of this host species were infested by *P*. *serrata* in Steglitz, but none of the five animals in Gatow (mid-P-exact-test: p = 0.047, Fig 3 and S2 Table). Moreover, lice were abundant on wood mice in Moabit and on bank voles and yellow-necked mice in Gatow and Steglitz, but completely absent from the latter hosts in Tegel (two-way-ANOVA: species p = 0.18, location p = 0.003, interaction p = 0.15).

**Fig 4:**
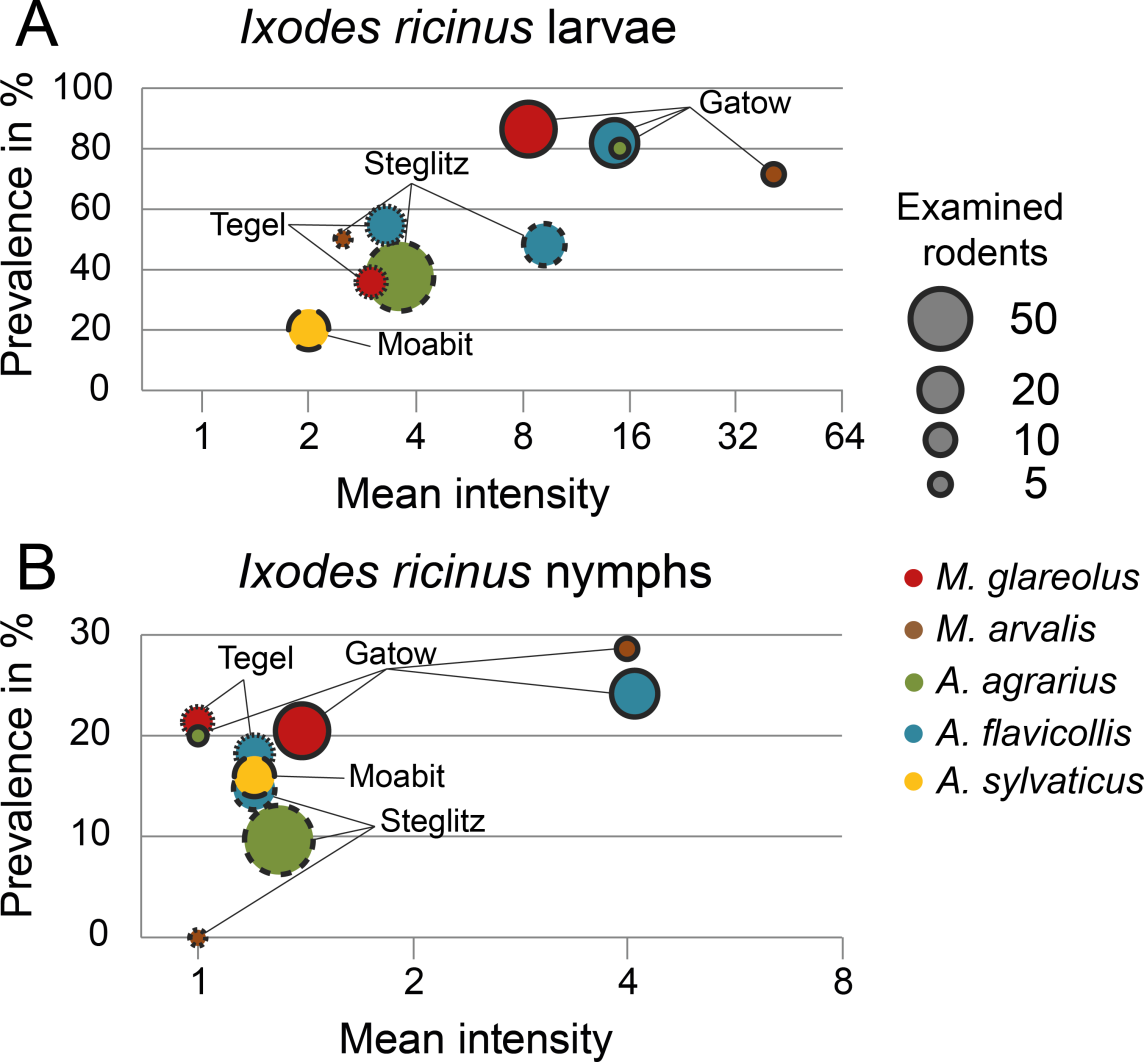
Prevalence and mean intensity of *I*. *ricinus* ticks. Bubble diagram showing prevalence and mean intensity of *I*. *ricinus* larvae (A) and nymphs (B) combined with the number of examined rodents (Bubble size) for five rodent species and the four trapping locations. Bubble colour indicates rodent species and the shape of margins the trapping location (also labelled). The two *M*. *agrestis* and the one *M*. *glareolus* captured in Moabit are not shown.

Hard ticks exhibited a low diversity with only three species, whereby *I*. *ricinus* was by far the most frequent, representing 99.2% of all rodent-attached ticks, followed by *Ixodes trianguliceps* (0.7%) and *Dermacentor reticulatus* (0.1%). *Ixodes ricinus* (S2 Fig) was the most prevalent arthropod species on rodents in Berlin, infesting 56% of the animals with an average of 9.4 ticks per infested rodent. The majority of individuals from this species represented the larval life stage (94%), the remaining were nymphs, while no adult ticks were observed. Prevalence and intensity of infestation with larval ticks did not differ between mice and voles (Fig 4A, LRA: voles-p = 0.782, S1C Table, NRA: vole-p = 0.320, S1K Table). On the species level, infested yellow-necked mice and common voles hosted significantly more *I*. *ricinus* larvae than did bank voles (NRA: *A*. *flavicollis*-RR = 1.84, p = 0.006, *M*. arvalis-RR = 3.58, p = 0.001, S1L Table). In contrast, intensity of infestation with larval ticks was lower in wood mice (same model, *A*. *sylvaticus*-RR = 0.23, p = 0.007). The location appears to be more important for the abundance of larval ticks. Host-associated *I*. *ricinus* larvae were most prevalent and numerous in Gatow followed by Steglitz and Tegel and were relatively rare at the most urban site in Moabit (Figs 3 and 4A). In Gatow, they were not only more prevalent with 84% of all rodents infested (LRA: Tegel-OR = 0.17, p<0.001, Steglitz-OR = 0.17, p<0.001, S1F Table), but also more numerous. On average, 13 *I*. *ricinus* larvae were observed on infested rodents, which was 6.5 fold higher than in Moabit (S2 Table) and, after correction for host species, 4.1 and 2.3 fold higher than in Tegel and Steglitz, respectively (NRA: Tegel-RR = 0.24, p<0.001, Steglitz-RR = 0.43, p = 0.002, S1L Table). In contrast, only 20% of the dominant wood mice at the Moabit site were infested and hosted only about two *I*. *ricinus* larvae. Nymphal ticks were generally less frequent and were observed on every 6^th^ rodent host. Infested *A*. *flavicollis* mice hosted on average 2.7 nymphs which is somewhat more than most other inspected rodent species (S2 Table), although this difference was only significant when compared to *M*. *glareolus* which were infested with 1.3 nymphs (NRA: *M*. *glareolus*-RR = 0.43, p = 0.02, S1M Table). In the same model, a trend of an increased intensity of infestation was recognised in Gatow with 2.8 nymphs compared to the other sites, where typically only one nymph was observed per rodent (Tegel-RR = 0.42, p = 0.052, Steglitz-RR = 0.43, p = 0.063; Fig 4B). With 18–28% prevalence, nymphs seemed to be slightly more frequent on rodents in Gatow and Tegel (periurban) than in Steglitz and Moabit (urban), where they reached 10–17% (Figs 4B and 3). However, this difference was not significant (LRA: urban-OR = 0.47 p = 0.178, S1G Table).

The encountered specimens of gamasid mites (Mesostigmata) belonged to the family Laelapidae and two families of non-parasitic, nest-associated mites, represented by three species of Parasitidae and two species of Ologamasidae. Most Laelapidae, represented by 13 species, are at least facultatively parasitic feeding on skin, lymph and blood. The only non-parasitic member of this family was *Hypoaspis sardoa* of the nidicolous subfamily Hypoaspidinae. The parasitic laelapid species were very frequent with 67% prevalence and were absent only from the two field voles. These mites parasitized bank voles with a 31% prevalence considerably less frequently than other rodent species which revealed a prevalence of 77–88% (Table 2 and Fig 3, LRA: *M*. *arvalis*-OR = 7.57, p = 0.019, *A*. *flavicollis*-OR = 5.77, p<0.001, *A*. *agrarius*-OR = 3.15, p = 0.063, S1D Table). The mean intensity of infestation was the highest in common voles with 13.4 (95% CI 8.4–25) gamasid mites per infested rodent compared to *M*. *glareolus*, *A*. *agrarius* and *A*. *sylvaticus*/location Moabit (undistinguishable due to collinearity), while *A*. *flavicollis* just missed significance (Table 2, NRA: *M*. *glareolus*-RR = 0.13, p<0.001; *A*. *agrarius*-RR = 0.37, p = 0.001; *A*. *sylvaticus*-RR = 0.38, p = 0.002; *A*. *flavicollis*-RR = 0.63, p = 0.09, respectively, S1N Table). The gamasid mite species exhibited marked host preferences. Within the genera *Laelaps* and *Hyperlaelaps*, which are strongly adapted to parasitism, *L*. *jettmari* occurred nearly exclusively on the striped field mouse, whereas *Laelaps agilis* infested animals of the rodent subgenus *Sylvaemus* comprising the yellow-necked mouse and the wood mouse. *Laelaps hilaris* (S2 Fig) and *Hyperlaelaps microti* virtually only parasitized the common vole (Fig 3 and S2 Table). With 35% overall prevalence, the most frequent gamasid species was the obligate parasite *H*. *sunci*, which clearly preferred *Apodemus* mice to voles (Fig 3). The related *Hirstionyssus (Echinonyssus) isabellinus* was strictly host-specific and represented the most abundant gamasid mite of bank voles. In contrast, the common, but facultative parasitic mite *Haemogamasus nidi* had a broad host range (Fig 3). The occurrence of the parasitic Gamasina was much more host-species-specific than location-specific. Indeed, the prevalence of infested yellow-necked mice was nearly the same in Steglitz, Tegel and Gatow or between bank voles in Tegel and Gatow. However, *Myonyssus gigas* infested yellow-necked mice solely in Tegel (Fig 3, mid-P-exact-test p<0.001, post-tests with Holm-correction: Gatow p = 0.015, Steglitz p = 0.015) and *H*. *isabellinus* (Fig 3) and *Hirstionyssus soricis* occurred only on bank voles in Gatow, although case numbers were too low for significance compared to Tegel (mid-P-exact-Test p = 0.067 and p = 0.76, respectively).

The prostigmatic mites of the Trombiculidae (chiggers) are only hematophagous as larvae, whereas the deutonymphal and adult life stages live as predators in the soil. In agreement with this life style, only larvae were found on the rodents. With only two species, *Hirsutiella zachvatkini* and the harvest mite *Neotrombicula autumnalis* (S2 Fig), the diversity was low. They occurred with only about 5% prevalence, however, with a high mean intensity of nearly five mites per infested rodent. They showed a marked preference for voles as compared to *Apodemus* mice (Fig 3 and Table 2, LRA: mouse-OR = 0.14, p<0.001, S1E Table). Trombiculidae exclusively infested sylvatic rodents in Gatow and Tegel. Since *H*. *zachvatkini* was exclusively present in Tegel (Fig 3), prevalence rates of Trombiculidae were higher in Tegel than in Gatow (same model, Tegel-OR 2.40, p = 0.029), and about eight times more trombiculid mite larvae fed on rodents in Tegel than in Gatow (NRA: Gatow-RR = 0.12, p<0.001, S1O Table).

Myobiidae are hematophagous stationary ectoparasites. Every species was specific to a particular host species (*Myobia agraria* on *A*. *agrarius* and *R*. *clethrionomys* on *M*. *glareolus*) or host genus (*Myobia muris-musculi* (S2 Fig), *Myobia multivaga* and *Radfordia lancearia* on *Apodemus* (*Sylvaemus*), *Radfordia lemnia* on *Microtus*). Prevalence rates of Myobiidae did not differ between rodent species or between mice and voles. The mean intensity of infestation was independent of the host species and trapping site and remained mostly within a small range between two and six mites per rodent (Table 2). In contrast, the Myobiidae infested mice in the periurban sites with 33–34% prevalence markedly less frequently than in the urban sites in Steglitz (59%) and Moabit (65%) (LRA: urban-OR = 6.53, p<0.001, S1H Table).

The astigmatic family Myocoptidae predominantly infested voles (Fig 3), except for seven mite specimens of *T*. *widawaensis* and *Criniscansor* sp., which were a rare finding on *Apodemus* mice and exclusively detected in Steglitz. The latter appeared to be an undescribed species from the genus *Criniscansor*. The most prevalent myocoptid species were *Myocoptes japonensis* (S2 Fig) and *T*. *tenax*, occurring on all three vole species. Together, they infested about 60% of all voles with a mean intensity of seven mites per infested animal (Table 2). The Myocoptidae were about two-times more prevalent in bank voles in Gatow than in Tegel (Mid-P-Exact-Test p = 0.008), although the infestation intensity was approximately the same at both periurban study sites (Mann-Whitney-U-Test: p = 0.65).

Listrophoridae are parasites feeding on organic material in the fur of rodents. Occasionally, they were found in high numbers with more than 100 mites per rodent host. In contrast to the Myocoptidae, the Listrophoridae preferred mice. The species *Afrolistrophorus apodemi* (S2 Fig) occurred on 39% of *Apodemus* mice, whereas the vole parasite *L*. *brevipes* only infested 15% of the voles, where it was only absent from *M*. *arvalis*. *Afrolistrophorus apodemi* was less abundant on *Apodemus* mice at the periurban sites Gatow and Tegel with 21% and 18% prevalence and a mean intensity of seven mites per mouse than on those in Moabit and Steglitz with 52% and 46% prevalence and an average of 14 mites per rodent (Fig 3, LRA and NRA within *Apodemus*: LRA: urban-OR = 4.64, p = 0.001, S1I Table, NRA: urban-RR = 2.96, p = 0.022, S1P Table). Surprisingly, the vole parasite *L*. *brevipes*, which infested 9 of 14 bank voles in Tegel was absent on 44 bank voles in Gatow (Fig 3, Mid-P-Exact-Test: p<0.001), although it was found on both field voles at this location.

Overall, rodents were infested by an average of 4.5 arthropod or 4.2 ectoparasite species. To test whether the diversity of arthropod species differed between hosts (both *Microtus* species combined), a D’Agostini-Pearson omnibus normality test was performed revealing that normality was only rejected for *M*. *glareolus* (p = 0.005). As species numbers per host also resembled normality in Quantile-Quantile-plots of the four other hosts, normal distribution was similarly assumed for the bank vole. The number of arthropod species differed significantly between rodents (one-way-ANOVA with Holm-corrected post-tests: p = 0.011), as *Microtus* voles hosted significantly more arthropod species (mean = 6.5) than did *A*. *agrarius* (4.4, p = 0.006), *A*. *flavicollis* (4.4, p = 0.010) and *M*. *glareolus* (4.0, p = 0.005). The difference to *A*. *sylvaticus* was not quite significant (4.68, p = 0.057). The highest arthropod diversity with 11 species was observed on two *A*. *flavicollis* and one *M*. *arvalis* in Steglitz and on one *M*. *glareolus* in Tegel. As an example, an individual *A*. *flavicollis* mouse was infested by the fleas *N*. *fasciatus* and *C*. *agyrtes*, the louse *P*. *serrata*, the tick *I*. *ricinus*, the gamasid mites *H*. *nidi*, *Eulaelaps stabularis* and *H*. *sunci* and the fur mites *A*. *apodemi*, *M*. *muris-musculi*, *M*. *multivaga* and *R*. *lancearia*.

### Seasonality

**Fig 5:**
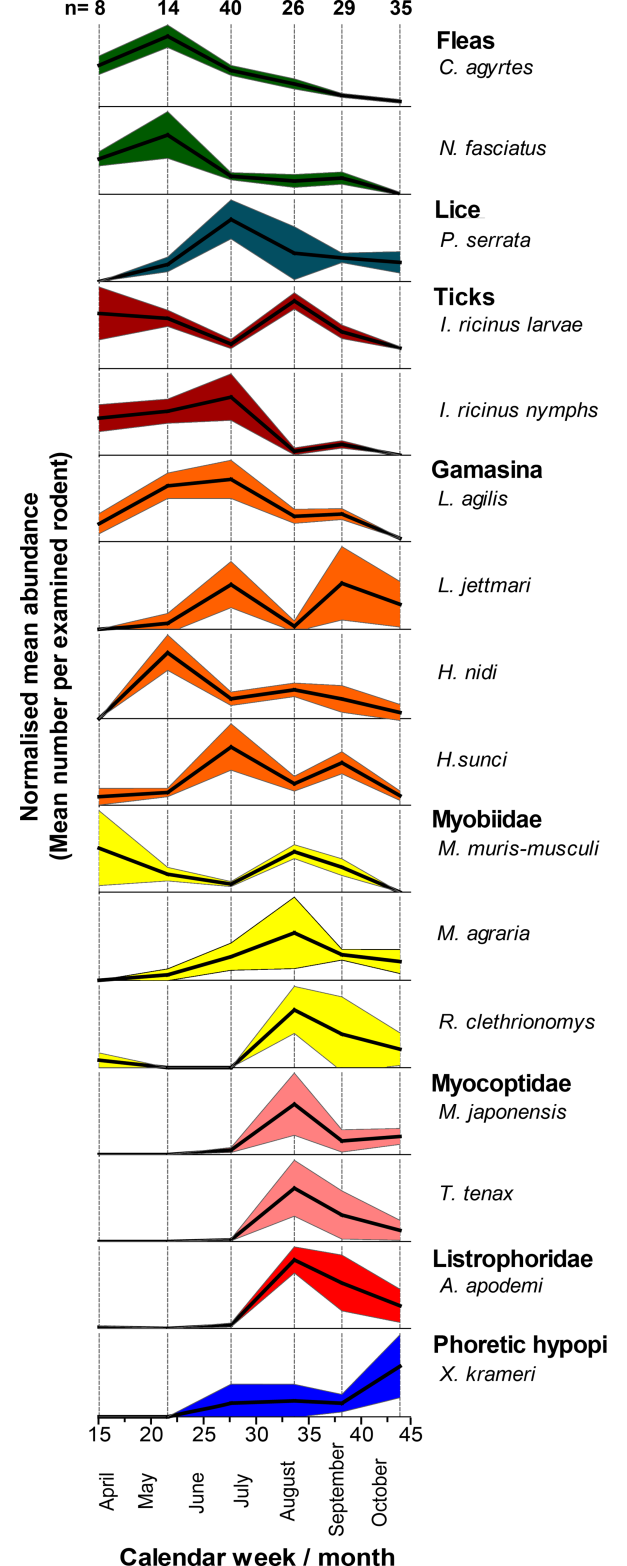
Seasonal abundance of common rodent-associated arthropods. The normalised mean abundance (thick solid line) per rodent host and standard errors of the mean (thin solid lines), normalised for trapping location and host species are shown for the most prevalent arthropod species on 152 rodents (three species from two study sites) from April to November 2011. The y axes were adjusted between species for better comparability of the time course, whereas abundance values were omitted, because of the lack of comparability between species due to normalisation. Dashed vertical lines indicate the mid time-point of trapping from every trapping block. Numbers of examined rodents are shown in the first row for every trapping block.

Most groups of ectoparasites occurred with specific patterns of seasonality. In the study period from April to October 2011, when the four study sites were trapped six times, fleas were most abundantly found in the fur of the rodents in late spring and early summer and became increasingly rare until the end of October (Fig 5). In contrast, *P*. *serrata* lice were quite rare in spring and reached their highest normalised mean abundance not before June/July, although the number of lice strongly varied between host individuals. Their peak of abundance occurred simultaneously with the peak trapping success for their hosts, *Apodemus* spp. mice (S1 Fig). Different patterns of seasonality were detected for larval and nymphal ticks. Whereas larval *I*. *ricinus* were detected continuously and frequently throughout the study period with only a slight peak in August, nymphs infested rodents at higher abundance until June/July and were nearly absent from rodents in August to October. No common pattern of seasonality was evident for the most prevalent species of gamasid mites. However, species strongly adapted to parasitism, such as *L*. *agilis*, *L*. *jettmari* and *H*. *sunci*, were most abundant in June/July with a second peak in September for the latter two species. In contrast, the facultative parasite *H*. *nidi* seemed to most abundantly infest rodents early in May. Some strongly adapted stationary parasite groups, such as the prostigmatic Myobiidae or the astigmatic Myocoptidae and Listrophoridae, occurred relatively late in the year starting only in June/July with a peak abundance in August. Also the nonparasitic, phoretic hypopial stages (deutonymphs) of *Xenoryctes krameri* increased in occurrence on the rodents in the early summer with an apparent peak abundance at the end of the study period.

### Regression model for the abundance of host-associated *I*. *ricinus* larvae

**Fig 6:**
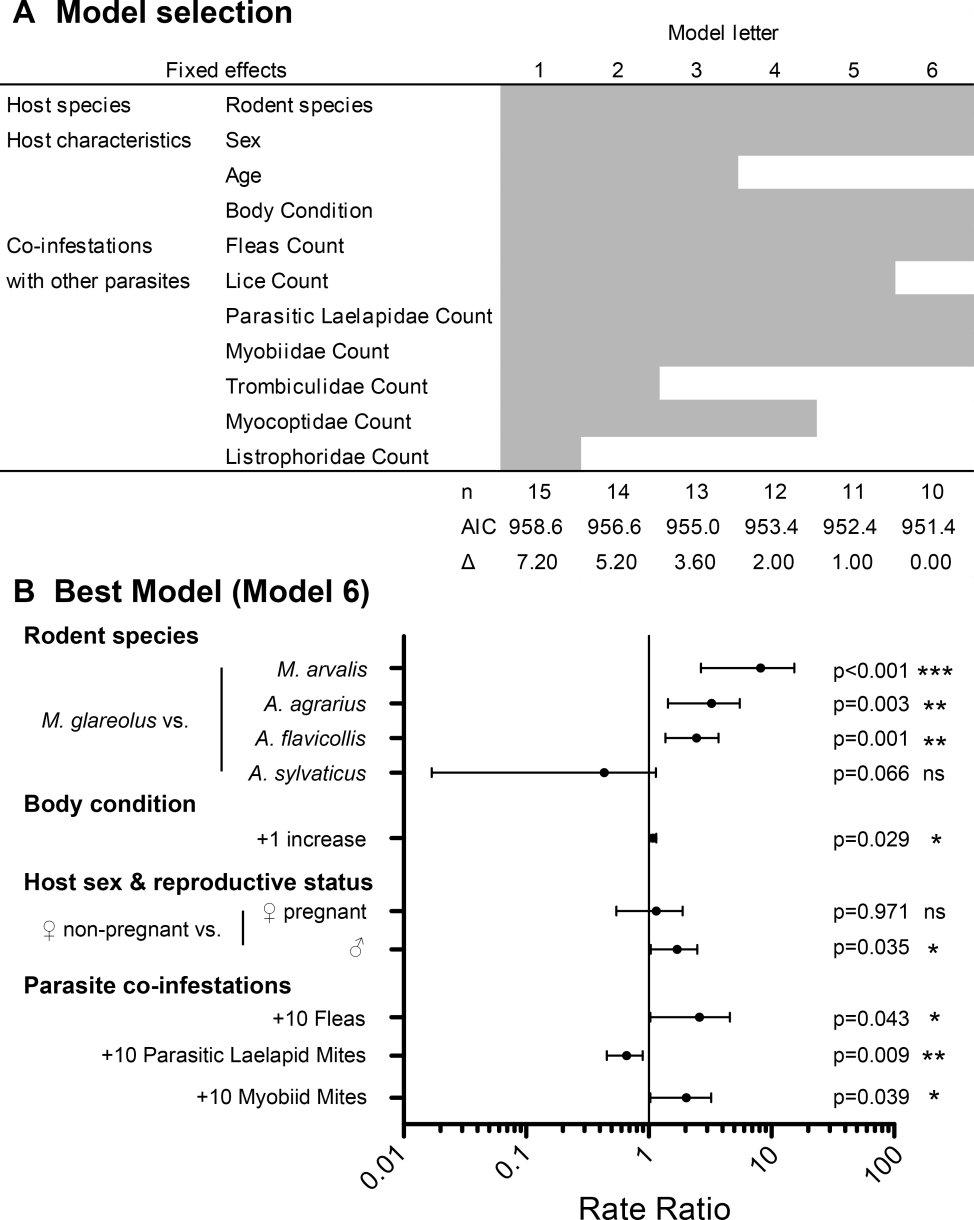
Parameters affecting number of host-associated *I*. *ricinus* larvae on wild rodents. (A) Model selection and (B) Forest Plot of negative binomial regression analysis of the count of *I*. *ricinus* larvae. (A) Analysis started with full model 1 including all the listed variables and was reduced by stepwise backwards variable selection to the best model 6. Number of variables (n), AIC values and difference of AIC to best model (Δ) are shown below. (B) Rate ratios with 95% CI for variables of model 6. The Y axis depicts additional counts (+) for metric parameters and reference levels in front of the other levels for categorical factors. Vertical line depicts rate ratio of 1 (no influence). * p<0.05; ** p<0.01; *** p<0.001.

After stepwise backward variable selection starting with the full negative binomial GLMM, model 6 appeared to be the best model (Fig 6A). No variable had to be removed because of collinearity, since VIFs were low (S3 Table). Six parameters significantly affected the number of larval ticks on trapped rodents: (I) the rodent host species, (II) the host sex including reproductive condition, (III) the host body condition, (IV) the number of fleas, (V) the number of parasitic Laelapidae mites and (VI) the number of myobiid mites co-infesting the rodents (Fig 6B). With all other variables remaining constant, tick infestation was significantly affected by the host species. *Myodes glareolus* voles were significantly less densely infested by *I*. *ricinus* larvae than were *M*. *arvalis* voles (6.4 times more, 95% CI 2.7–15.3, p<0.001) and *A*. *agrarius* (2.8 times more, 95% CI 1.4–5.5, p = 0.003) and *A*. *flavicollis* mice (2.2 times more, 95% CI 1.4–3.7, p = 0.001). The biological host characteristic age (estimated from the z values of eye lens weight) was poorly correlated with the tick count and was hence excluded from the final model. In contrast, body condition (the nutritional status) and host sex combined with reproductive condition significantly affected the larval *I*. *ricinus* count. Rodents that had a better body condition hosted more tick larvae and although tick abundance did not differ between pregnant and non-pregnant females, male rodents hosted 1.6 times (95% CI 1.03–2.48, p = 0.035) more larval ticks than did non-pregnant females. The abundance of most of the ectoparasite groups did not affect the number of ticks on mice and voles and were excluded from the best model selected. However, the counts of fleas, parasitic Laelapidae mites and Myobiidae mites influenced the level of tick infestation. Theoretically, each additional mite on a rodent reduced the number of larval ticks by 4.5% (95% CI 1.2–7.6%, p = 0.009). In contrast, but with low significance, every flea and every myobiid mite increased the number of *I*. *ricinus* larvae by 8.0% (95% CI 0.3–16.4%, p = 0.043) and 6.2% (95% CI 0.3–12.4%, p = 0.039), respectively.

## Discussion

Small mammals are essential hosts for the immature life stages of the most important arthropod vector of pathogens in Central Europe, the hard tick *I*. *ricinus*. Furthermore, a large number of other arthropods, such as fleas, lice and numerous mite species, are associated with these mammals. In most studies about ectoparasites of wild rodents, only single arthropod species/groups were investigated. Moreover, the majority of publications focuses on qualitative data, such as species lists and descriptions of new species. Particularly, data on mites are scarce.

This study represents the first comprehensive investigation on the diversity, prevalence and intensity of rodent-associated arthropods on wild mice and voles, comparing these aspects in an urban/periurban context. The variety of rodent species and ectoparasite species differed significantly among the four trapping sites in Berlin. The trapping location affected not only the rodent trapping success, but also the rodent species. The urban Botanic garden in Steglitz with its old trees and a marked shrub and ground vegetation provided ideal conditions for the striped field mouse *A*. *agrarius*. The sylvatic species yellow-necked mouse (*A*. *flavicollis*) and bank vole (*M*. *glareolus*) prefer old forests [1, 71] and were thus the most abundant species in the forest sites Tegel and Gatow. In Tegel, fewer rodents were observed than expected, possibly because at least in parts of that study site concurrent rodent control measures were undertaken. The trapping site in Moabit, an inner-city backyard, was the most urbanized location, with the wood mouse *A*. *sylvaticus* constituting the dominant rodent. In contrast to its name, it is a rather euryoecious species [2, 72] and able to colonize anthropologically disturbed areas [73] in close proximity to humans.

Only three reports from non-Russian Europe investigated the whole array of rodent-associated arthropods on the host species investigated here (Table 3). One of them is so old that comparisons may not be drawn due to new and re-descriptions [74]. In a study on larger arthropods, small fur mites and other Astigmata infesting the yellow-necked mouse and the bank vole in Poland, the number of examined hosts was not reported, small mite groups were not determined to species level and rare species were likely overlooked [75]. The sole directly comparable studies are those by Ryszard Haitlinger, who examined numerous small mammals, including 3,307 specimens of the rodent species examined here [76–84] at numerous sites in Poland over the last 20 years. Overall, he differentiated approximately 150 arthropod species. The 20 trapping sites in the northern Lubuskie province, Poland [80], were the closest to Berlin with 90–170 km distance. Here, he determined a similar species richness of 69 arthropod species, 43 of which are parasitic, on a comparable number of 277 mice and voles. Haitlinger found fewer small arthropod species (Myobiidae, Myocoptidae). Because of their small body size, these species are easily overlooked, and the examination method presented herein seems to be more sensitive. Nevertheless, all myobiid and myocoptid species (except the undescribed *Criniscansor* sp.) were detected by Haitlinger in other regions in Poland, probably only when they densely infested their hosts. However, true differences in fur mite diversity between the rural area in Poland and Berlin cannot be excluded. In accordance with most other studies from Europe [68, 69, 85–90], Haitlinger determined more flea, trombiculid mite and non-parasitic gamasid mite species than recorded herein (Table 3). These arthropods exhibit a poor association with the mammalian host: First, the majority of flea species detected on the rodents were nest-associated fleas, which spend most of their life in the nest and only the adults temporarily parasitize the host. In addition, these species exhibit low host-specificity. Second, Trombiculidae are predatory mites, which are parasitic only in their larval stage, so-called chiggers. They have a wide host array feeding on numerous terrestrial vertebrates [35]. Third, the nidicolous, non-parasitic Gamasina feed on other arthropods, nematodes and dead organic material from mammals. The occurrence of these three groups, therefore, depends rather on habitat preferences than on the rodent host. As rodents were trapped by Haitlinger [80] in rural, sparsely populated areas, these natural habitats appear to facilitate higher species richness in arthropods with lower association to rodent hosts compared to highly fragmented urban wooded and non-wooded areas examined herein. In contrast, the diversity and composition of highly specialised stationary ectoparasite species was very similar between the present and the mentioned studies from Europe and distinctly determined by the presence of the respective host species. Urbanisation may influence the presence of highly adapted rodent ectoparasites only if it affects the presence and abundance of the specific rodent host species.

**Table 3:** Number of species of different rodent-associated arthropod groups infesting the rodent species of the present study from similar studies from non-Russian Europe.

Reference		Elton et al., 1931 [74]	Willmann, 1952 [87]	Stammer, 1956 [69]	Mahnert, 1971abc [85, 91, 92], 1972 [86]	Artz, 1975 [68]	Ambros, 1984 [88], Kovacik, 1984 [89], Dudich, 1984 [90]	Harris et al., 2009 [75]	Haitlinger, 2009 [80]	present study
Locality		England	Germany/ Poland	Germany	Austria	Germany	Slovakia	Poland	Poland	Germany
		Number of examined hosts								
*M*. *glareolus*		281a^a^	150	144	203	98	209–219^e^	? ^f^	97	59
*M*. *arvalis*		-	59	107	2	219	7–9^e^	-	35	11
*M*. *agrestis*		368^a^	2	51	46	19	-	-	7	2
*A*. *agrarius*		-	36	3	-	-	9	-	92	77
*A*. *flavicollis*		-	245	25^b^	79	311	25–40^e^	? ^f^	36	82
*A*. *sylvaticus*		988^a^	178	198	16	44	-	-	10	25
Total		1637^a^	670	528	349^c^	691	9	? ^f^	277	256
		Number of arthropod species								
Diptera		0	-	1	-	0	-	0	0	0
Coleoptera		1	-	0	-	1	-	0	0	0
Siphonaptera		11	-	11	20^d^	14	12	9	12	10
Anoplura		2	-	-	4	3	3	1	4	4
Ixodida		1	-	-	3	2	1	2	2	3
Gamasina	parasitic	7	14	-	13	-	10	6	14	13
	non-parasitic	7	7	-	15	-	7	1	18	6
Myobiidae		0	3	-	-	-	-	1	2	6
Trombiculidae		1	4	3		-	6	1	6	2
Pygmephoridae		0	4	7	2	-	-	0	1	1
Myocoptidae		0	2	-	-	-	-	1^g^	1	4
Listrophoridae		1	1	-	-	-	-	1^g^	2	2
other parasitic		1	0	-	0	-	-	0	0	5^h^
other non-parasitic		3	0	9	1	-	-	1^g^	7	7
Total		35	35	31	58	20	39	24	69	63

Only the study closest to Germany from Ryszard Haitlinger is illustrated. Some studies investigated further host species which are not displayed here. Hyphens indicate (presumably) not investigated arthropod groups

^a^ Number of examined hosts not clear and different between arthropod group

^b^ 25 *A*. *flavicollis* according to the lists but 35 according to the text

^c^ Three *Apodemus* sp. specimens not determined to species level

^d^ 21 if two subspecies of *Doratopsylla dasycnema* are considered

^e^ Number of examined hosts differed between arthropod groups

^f^ Number of examined hosts are not reported. Only *M*. *glareolus* screened for fur mites and other Astigmata

^g^ Arthropod group not determined to species level and probably include more species

^h^ all parasites in nasal cavity or skin

Concerning the quantity of infestations, there are no comprehensive data on the whole array of arthropods for comparison of the apparently high prevalence (99%) and mean intensity (16 specimens per host) in the present study. However, this may be common in nature or even low, as in a longitudinal study from south-central Sweden [93], tick larvae alone infested 100% of rodents with mean intensities of 34 (*M*. *glareolus*, n = 106) to 68 larvae (*A*. *flavicollis*, n = 31). Our live trapping method allowed us to detect the majority of arthropods living in the fur of rodents. Nevertheless, fleas, in particular, are known to leave the host rapidly during disturbance [94], although in an experiment only about 5% of the fleas left the live trap when a rodent was trapped for 10 hours [68]. Since also trap contents (cotton, apple, faeces) were screened, it can be expected that only a small number of fleas and other arthropods has been lost. In contrast, it cannot be excluded that individual ground-dwelling arthropods were examined, which entered the traps independently from the trapped host.

The quantity and species diversity of rodent-associated arthropods strongly depended on the rodent species and/or trapping location. Similarly, Timm [95] recognised three primary categories of ectoparasites of mammals: (1) the host-specific, (2) the habitat-specific and (3) the cosmopolitan parasites. Voles (family Cricetidae), represented by three species, were much more frequently infested by trombiculid larvae and Myocoptidae than mice of the genus *Apodemus* (family Muridae). In contrast, mice more often hosted lice (most of all *A*. *agrarius*), Listrophoridae and the infestation intensity with fleas was higher than on voles. Hard ticks, gamasid and myobiid mites, on the other hand, revealed comparable prevalence and infestation intensities among the different rodent families. The reason, although not the cause, for differences in the quantitative occurrence of stationary parasitic groups, is that mice and voles are infested by different host-specific species, such as Anoplura, Myocoptidae and Listrophoridae. Differences may be caused by the width of species-specific niches of the arthropods concerning texture, density and diameter of hairs [96] as well as grooming behaviour of mice and voles.

Fleas, chiggers and ticks were not markedly host-specific parasites. The reason for the higher number of fleas per mouse may be their larger body surface with their relatively long extremities compared to the rather compact vole body. Hence, fleas were most numerous on yellow-necked mice, the largest rodent in our study. In contrast, chiggers probably preferred the more subterranean life of voles to that of mice. Hard ticks, most notably *I*. *ricinus* larvae and to a smaller extent nymphs, occurred with an overall similar prevalence on all host species trapped at the same location. However, on the yellow-necked mouse, both life stages were more numerous than on the bank vole, often occurring syntopically, confirming earlier observations in Berlin [8] and in south-central Sweden [93]. In the latter study, larvae and nymphs infesting *A*. *flavicollis* appeared to have a higher feeding success compared to those ticks infesting *M*. *glareolus* [93]. The yellow-necked mouse, therefore, might be somewhat more suitable as host for this tick species. The lower prevalence of subadult ticks on *A*. *sylvaticus* may be explained by the trapping location Moabit (see below). Differences in prevalence and infestation intensity of the mainly host-specific gamasid and myobiid mites were not pronounced among rodents, because almost every host species was infested by a specific mite species. Only the bank vole was less frequently infested by Gamasina presumably because its specific *Laelaps* species, *L*. *clethrionomydis*, was absent, as it primarily occurs in submontane and montane regions [97].

The trapping location was an equally important factor for the occurrence of different parasitic groups on the rodents. The backyard in Moabit in the city centre of Berlin was the most urbanized location and the surrounding habitat was highly fragmented. Periodic and temporary parasites, such as fleas, ticks and Trombiculidae live most of their life span off the host. They strongly depend on environmental parameters and,require undisturbed habitats. Thus, they were infrequently found on rodents at this urbanized location. For nest-associated fleas and especially their juvenile stages, the properties of the host’s nest, such as temperature and relative humidity of 70–80% [18] are more important than host characteristics [86]. Similarly, *I*. *ricinus* larvae require high levels of relative humidity [98]. As the different stages of this tick prefer various hosts, their further development depends on access and habitat quality for these vertebrates. The trombiculid mites require high humidity [99] and undisturbed ground fauna where they live as predators in their deutonymphal and adult stages. In contrast, stationary parasites, such as Myobiidae and Listrophoridae, closely adapted to the host fur, were very prevalent and numerous on mice in Moabit. Since *A*. *sylvaticus* was almost the only rodent species at the Moabit site and absent from the other sites, this collinearity renders it impossible to statistically differentiate the effects of urbanisation and host species. However, because of the close phylogenetic relation to *A*. *flavicollis*, these differences are unlikely to be particular features of the wood mouse.

The sites in Tegel and Gatow were comparable periurban forest habitats situated at the periphery of Berlin and characterised by little fragmentation and anthropogenic impact in contrast to the Moabit site. At these locations, temporary or periodic parasites, especially chiggers, were more prevalent and numerous than at any other site. Also, subadult *I*. *ricinus* infested more rodents with higher intensities than in the more urbanized locations. The differences between the two forest sites were mainly on the species level for some frequent parasites (e.g. *H*. *zachvatkini* (Trombiculidae), *L*. *brevipes* (Listrophoridae), *H*. *isabellinus* (Gamasina)), whereas sucking lice were completely absent in Tegel. In general, louse species showed a very peculiar and focal occurrence. Although stationary parasites, they were not substantially more abundant in urban sites and they were occasionally absent from rodents in particular locations. Whether this observation results from differing microclimate, predators, or simply from founder effects in these particular habitats remains to be examined.

The Botanic Garden in Steglitz revealed an intermediate composition of rodent ectoparasites. Although in the centre of an urban area, it is characterised by diverse and structured vegetation with old trees and constituted a habitat even for the sylvatic yellow-necked mouse. A quantity of ticks and fleas comparable to that at the forest sites was found, but no chiggers and higher prevalence of stationary fur mites, such as Myobiidae and Listrophoridae, similar to the urban backyard Moabit.

Seasonal differences in the abundances of rodent-associated arthropods are expected in temperate regions and must be interpreted in respect to host population dynamics. The bank vole and the yellow-necked mouse are both mainly bivoltine, with the first litter from overwintered females in spring and the second from these females together with their progenies in early summer [71, 75, 100]. Therefore, the greatest recruitment of “new” rodent hosts occurs in July/August resulting in a dilution of parasite numbers on individual hosts [75], which may be most noticeable for parasites with long generation times. In the present study, two flea species, including *C*. *agyrtes* as the second most prevalent parasite, both occurred throughout the study period and showed a unimodal occurrence with peak abundance on the rodents in early spring. This was similarly observed for *C*. *agyrtes* in Poland [101], but in other studies a second peak in late summer/autumn was observed [18, 75]. The sucking louse *P*. *serrata* was most abundant in summer which is consistent with the occurrence of *Hoplopleura edentula* and *Hoplopleura acanthopus* in Tyrol, Austria (too few data for *P*. *serrata*) [92], *H*. *edentula* in Poland [101] and other studies [102, 103]. Lice produce only one generation of offspring during the summer [92, 104] presumably because of the higher fecundity during moderate temperatures [105] and the higher density and activity of the rodent hosts [102]. Similar to the present observations, the seasonality of rodent-associated *I*. *ricinus* larvae is often described as bimodal with peaks in early summer and autumn [106, 107] and near absence in winter. In some other studies, the depression in midsummer was missing [8, 108]. Theoretically, the reduced infestation of rodents with larval ticks in July may have three reasons: First, the increase in rodent abundance at this time may result in fewer ticks feeding on individual rodents [8]. Second, a probable bimodal recruitment of “new” *I*. *ricinus* larvae may derive from overwintering eggs or overwintering engorged females in spring and from engorged females of the same year in late summer. Third, Randolph and Storey [98] demonstrated experimentally that *I*. *ricinus* larvae quest for hosts in the ground vegetation during periods of low saturation deficits as an index of humidity, whereas nymphs move to vantage points high above the ground. In contrast, during periods of high saturation deficits, larvae remain inactive, whereas nymphs continue to quest for hosts low in the vegetation, where they are more likely to encounter small mammals [98]. Since saturation deficits were highest in the summer in Berlin (S3 Fig), this may explain why larvae were rarely detected on rodents in summer, while nymphs parasitized rodents most abundantly. Parasitic gamasid mites showed differences in seasonality depending on the species. The closely host-associated mites were most abundant in summer and autumn, which is also described for *L*. *agilis* in southern Sweden [109]. In Poland, only one peak in July was observed [75]. The facultative parasite *H*. *nidi* most abundantly infested rodents in early summer. In the Swedish study, seasonal occurrence of this mite differed between trapping sites, but all gamasid mites reproduced throughout the year [109]. Only little is known about seasonality of the very small fur mites and astigmat hypopi which use rodent fur for phoresy. Surprisingly, all of them revealed a similar pattern being most abundant in August (fur mites) or October (phoretic hypopi), but were nearly absent until July. This may be a methodological bias because higher awareness presumably increased the sensitivity of detection of these small mites towards the end of the study. In contrast, fur mites were abundant throughout the year on bank voles in Poland [75], whereas *Listrophorus* was slightly less abundant in summer.

Because of their mobility, broad host array and the ability to penetrate human skin, only fleas, hard ticks, chiggers and laelapid mites may potentially constitute a direct or indirect zoonotic and public health relevance. Specimens of these groups occurred on 95% of the rodents in Berlin with an average of 12 arthropod specimens per rodent. However, only a small number of these species have actually been recorded to infest humans. Rodent fleas are mainly nest-associated and humans generally do not come in close contact with rodent nests [18].

Of the flea species found in the present study, only the squirrel or dormouse flea *Monopsyllus sciurorum* and *N*. *fasciatus*, which primarily infests rats and house mice, have been described to attack humans [18]. Both species have a wide host array and whereas *M*. *sciurorum* was rare, *N*. *fasciatus* infested 18% of the rodents in our study, mainly *Apodemus* mice. The vector role of *M*. *sciurorum* is unknown, but *N*. *fasciatus* is able to transmit the tapeworms *Hymenolepis diminuta* and *Rodentolepis nana* (syn. *Hymenolepis nana*) [105] and is a competent vector for *Yersinia pestis* and *R*. *typhi* [110]. Nevertheless, it is unlikely that people ingest fleas infected with these tapeworms [105] and infections in humans are exceedingly rare in Central Europe [111, 112]. The plague is no longer endemic in Europe and the role of these fleas in the transmission of *R*. *typhi* is presumably poor [110].

Of the chigger species, at least the harvest mite *N*. *autumnalis*, which was abundant at periurban sites, infests humans causing scrub itch and pruritic dermatitis [113]. The hard tick *I*. *ricinus* abundantly parasitized rodents in all study sites in Berlin. As long as they are attached to rodents, both ectoparasites do not pose a direct risk of infestation for humans, but these hosts promote the development of the next generation (chiggers) or life stage (ticks) of ectoparasites in proximity to humans. More importantly, rodents are reservoirs for tick-borne pathogens and may maintain the transmission cycles of these pathogens, constituting a risk of contact with infected nymphal or adult ticks [7].

Reports regarding the infestation of humans with gamasid mites from the family Laelapidae are very rare and mostly doubtful. An often cited case of mite dermatitis caused by *Haemogamasus pontiger* in soldiers in England during the Second World War was challenged by Halliday [114], as it was more likely caused by *Pyemotis* mites. Likewise, reddish papulous dermatitis caused by *Androlaelaps casalis* on humans in England (personal communication from Evans in Baker et al. [115]) and in Israel derived from contacts with rat and pigeon nests [116] were reported and the mite was shown to feed on droplets of human blood [117]. In contrast, the latter author suggested that the mite was unable to penetrate vertebrate (including human) skin which was confirmed by Lesna et al. [118]. The species *Laelaps nutalli*, *Laelaps echidninus* and *Androlaelaps fahrenholzi* are unable to feed on intact skin, but feed on abraded human skin [119]. Nevertheless, infestations of humans are mainly conceivable after rodent control measures, when the natural hosts have been eliminated. However, the peridomestic rodent species trapped in the present study usually do not live inside houses.

The direct zoonotic risk of arthropods associated with peridomestic rodents is low in Berlin because these rodent species rarely come in close contact with humans and the majority of ectoparasite species has never been reported to infest people. Apart from *N*. *fasciatus*, typical rodent-borne zoonotic arthropods, such as the tropical rat mite *Ornithonyssus bacoti* and *Liponyssoides sanguineus*, a vector for *Rickettsia akari* [120], were absent. These ectoparasites are primarily associated with rats and house mice that live in close proximity to humans, but are rarely found on other mice and voles.

However, several arthropod species detected herein may be important for the circulation of pathogens in the rodent population. DNA of two zoonotic *Bartonella* species was detected in *N*. *fasciatus*, *C*. *agyrtes* and *Megabothris turbidus* fleas from rodents in Saxony [121], and DNA of *Rickettsia* in *C*. *agyrtes* fleas [12], *H*. *zachvatkini* and *N*. *autumnalis chiggers*, and *L*. *agilis* and *H*. *nidi* mites (Gamasina) in Slovakia [11]. Further pathogens were isolated and identified by culture, such as *Francisella tularensis* from *H*. *nidi*, *L*. *hilaris* and *H*. *isabellinus*, *Coxiella burnetii* from *H*. *nidi*, *Haemogamasus hirsutus*, *E*. *stabularis* and *A*. *fahrenholzi* and the TBE virus from *H*. *nidi*, *H*. *hirsutus*, *H*. *isabellinus*, *E*. *stabularis* and *A*. *fahrenholzi* [10]. Whether these findings only reflect the gut content of the arthropods containing blood from infected rodents or whether the mites are vector-competent remains to be examined in appropriate transmission experiments. However, at least *E*. *stabularis* is able to become and remain experimentally infected with the TBE virus and to transmit it to rodents [122]. If the mentioned transmission of pathogens by *C*. *agyrtes* would be possible, this abundant flea species, infesting every second rodent in the present study, may have a strong impact on the transmission within the rodent reservoir.

Infected ticks constitute a major health risk for people and the multivariate regression analysis identified significant parameters affecting the abundance of larval ticks on peridomestic rodents. Apart from the trapping location and season, the host species was the most important factor. The broad host array of this tick suggests that differences in the quantity of ticks on rodent species may depend on the frequency of tick-host encounters. The infestation intensity of rodents with ticks is positively correlated with the distance rodents migrate between successive captures [123]. *Apodemus* mice have larger home ranges than bank voles [124] and, therefore, should encounter more ticks. Also, different levels of tick mortality during the blood meal may result from different grooming behaviour and immune reactions [125]. Male rodents hosted more *I*. *ricinus* larvae than non-pregnant females. Male-biased parasitism is a commonly observed phenomenon for many mammals and is often attributed to differences in body size, behaviour, such as grooming or home range, and physiology, such as the immunosuppressive effect of testosterone (see Harrison et al. [126] and Marshall [105] for review). Although body size increases with age, this variable had only a minor effect on the tick infestation in the present study. Behavioural reasons for a male bias are more likely, since the home ranges of male *Myodes*, *Microtus* and *Apodemus* rodents are larger than those of conspecific females especially during the breeding season (reviewed by Tälleklint and Jaenson [93]) and consequently they should encounter more ticks. The pregnancy of females did not affect the tick count, although an energy-consuming active reproductive status may reduce the immune status and affect the feeding success of ticks. Malnutrition is known to impair immunity and resistance to parasites [127], but, in contrast, a better body condition was surprisingly associated with a slight increase in the abundance of *I*. *ricinus* larvae. Limitation of food presumably did not really occur during the study period since rodent densities were moderate. In a study in Hesse, Germany, a regression analysis with a forward variable selection was performed on a comparable number of mice and voles [128]. They similarly identified location, season, host species and body mass as important factors for tick infestation on rodents. In contrast, they found that age had an impact as well as to some extent rodent density, relative humidity and vegetation cover, whereas host sex failed to improve their model.

It was analysed whether ectoparasitic co-infestations affect tick abundance on rodent hosts considering all the co-factors in the regression model. Fleas and mites from the family Myobiidae were positively associated with larval tick abundance, but the significance was low. In contrast, an increasing abundance of co-infesting ectoparasitic gamasid mites of the family Laelapidae was significantly correlated with a reduced abundance of *I*. *ricinus* larvae on mice and voles. Some mites are known to predate on ectoparasites in the fur of mammals, such as the Cheyletidae *Cheyletiella parasitivorax*, *Chelacaropsis moorei* and *Hemicheyletus* spp. on Listrophoridae and other parasitic arthropods [113, 129]. But according to Samish and Alekseev [130], mites were reported only once to feed on host-attached ticks, namely the chigger *Parasecia gurneyi* on larval *Ixodes scapularis* [131]. Parasitism in laelapid mites derives from predation on arthropods (subfamily Hypoaspidinae) and species of the family represent all stages from facultative to obligate blood-sucking parasitism [113]. The normal diet of at least *A*. *fahrenholzi*, *E*. *stabularis* and *H*. *nidi* contains arthropods. In laboratory feeding tests, *H*. *ambulans*, *L*. *echidninus* and *A*. *fahrenholzi* fed on blood-filled sucking lice [113]. Ticks, which feed on their hosts for several days, can hardly defend themselves and may be an easily accessible prey. Increased grooming due to mite infestation may result in simultaneous removal of feeding ticks. Competition for space, release of toxic products by feeding mites, or an interaction of both ectoparasites via the immune system are further potential explanations [132]. Although the immune responses towards feeding gamasid mites are poorly studied, they presumably initiate type 2 T helper cell responses, as ticks do in the skin of rodents. Immune reactions against mites therefore may impair tick feeding as well. Although infections with intestinal endoparasites in laboratory mice failed to affect the success of tick infestation [67], interspecific local immune reactions in the same compartment, the skin, may be conceivable. Whether the negative relationship of the abundance of laelapid mites and *I*. *ricinus* larvae is caused by direct interactions between the parasites or via the rodent host remains to be examined experimentally. However, the abundant co-infestations of peridomestic rodents with parasitic Laelapidae may influence the suitability of rodents as hosts for ticks and their reservoir competence for tick-borne pathogens.

## Supporting information

S1 Fig Seasonal rodent trapping success at trapping sites. For every rodent species, the numbers of trapped animals are shown for every trapping week (three consecutive nights) and for four different study sites. Dashed line indicates the turn of the year.
(PDF)

S2 Fig **Light micrographs (A, D, E, G-K) and photographs (B, C, F) of diverse rodent-associated arthropod species collected from mice and voles from Berlin.** A *Ctenophthalmus agyrtes* (Siphonaptera) male, B *Polyplax serrata* (Anoplura) infesting the ear margin of *A*. *agrarius*, C *Ixodes ricinus* (Ixodidae) larvae and nymphs infesting neck of *A*. *flavicollis*, D *Laelaps hilaris* (Laelapidae) female, E *Neotrombicula autumnalis* (Trombiculidae) larva, F *Hirsutiella zachvatkini* larvae infesting ear of *M*. *glareolus*, G *Myobia muris-musculi* (Myobiidae) female, H *Myocoptes japonensis* (Myocoptidae) female, I *Afrolistrophorus apodemi* (Listrophoridae) female, J phoretic hypopus (deutonymph) of *Acarus nidicolous* attached to sternal plates of *Megabothris turbidus* (Siphonaptera), K phoretic hypopus of *Glycyphagus hypudaei* attached to a hair of *M*. *glareolus*, scale bars 0.1 mm, specimens in A and D were cleared in potassium hydroxide.
(PDF)

S3 Fig Course of saturation deficit in an urban park in Berlin Steglitz in 2011. Daily means of minute values of saturation deficit calculated from relative humidity and temperature (table 10 in Deutscher Wetterdienst, 1998). Data were measured by a climate station (THIES Clima) at a height of 2 m every day of the year 2011 and were provided by the Institute of Meteorology, Freie Universität Berlin. Line chart (red) is depicted together with an order 4 polynomial trend line (black).
(PDF)

S1 Table Regression analyses for modelling presence/absence (= prevalence) and count (= intensity of infestation) of ectoparasites on peridomestic rodents from Berlin as dependent variable. Rodent host species (6 levels) or family (2 levels) and trapping location (4 levels) or location category (2 levels) were used as independent variables. Odds Ratios (OR) for logistic regression (left panel) or Rate Ratios for negative binomial regression (right panel) are shown together with 95% CI. P-values are only shown for one reference level of interest compared to the other levels within the same variable, since correction for multiple testing would drastically increase p-values and hence reduce significances. * p<0.05, ** p<0.01, *** p<0.001, inf: infinite, n.a.: not applicable because of total collinearity between *A*. *sylvaticus* and the trapping location Moabit.
(DOCX)

S2 Table Distribution of arthropods on wild rodent species. Total number and sex ratio (male: female) of parasites as well as number of infected rodents, prevalence and mean intensity for six rodent species are shown for every arthropod species. The number of examined rodents and the number of male/female are given below the species name. The last column shows values for the sum of all rodent species. Capital letters next to the families indicate higher arthropod taxa: Si Siphonaptera (fleas), Ph Phthiraptera (lice), Ix Ixodida (ticks), Ga Gamasina (Mesostigmata), Pr Prostigmata, As Astigmata. n: Number of arthropods. Hyphens indicate absence of arthropods. No: Number of infested rodents. P [%]: Prevalence in %. mI [n]: mean intensity = mean number of parasites on infected rodents. max: highest arthropod intensity. n.d.: no adults observed or not determined.
(DOCX)

S3 Table Variance inflation factors of metric and integer variables in regression model for the abundance of host-associated *I*. *ricinus* larvae (Fig 6). All variables have a variance inflation factor below a threshold of 5 and therefore can be considered to have low collinearity with the other variables.
(DOCX)

S1 File Original table with raw data of rodent trapping and arthropod identification. Rows in the sheet “Data” represent trapped rodent individuals with data on rodent taxonomy, trapping, rodent body measures and arthropod counts. Shaded rodents were released or escaped and were not further investigated. The sheet “Codebook” lists the description and scale levels of every column.
(XLSM)
