# Supplementary figures and images for: Factors associated with diversity, quantity and zoonotic potential of ectoparasites on urban mice and voles
Source: PLoS One. 2018 Jun 25;13(6):e0199385. doi: 10.1371/journal.pone.0199385 (PMC6016914; doi:10.1371/journal.pone.0199385)

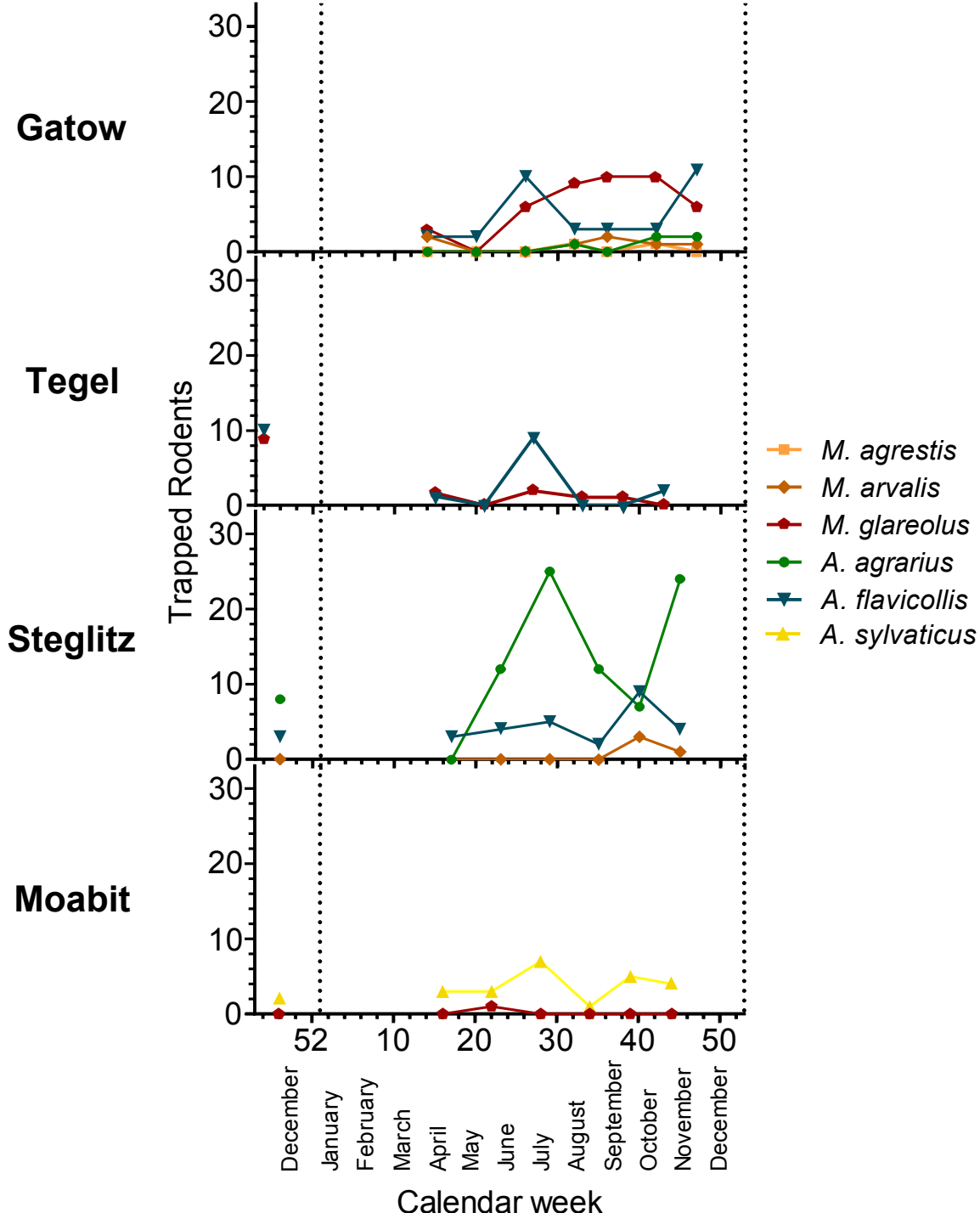

Supplement: S1 Fig — For every rodent species, the numbers of trapped animals are shown for every trapping week (three consecutive nights) and for four different study sites. Dashed line indicates the turn of the year. (PDF) [file pone.0199385.s001.pdf]

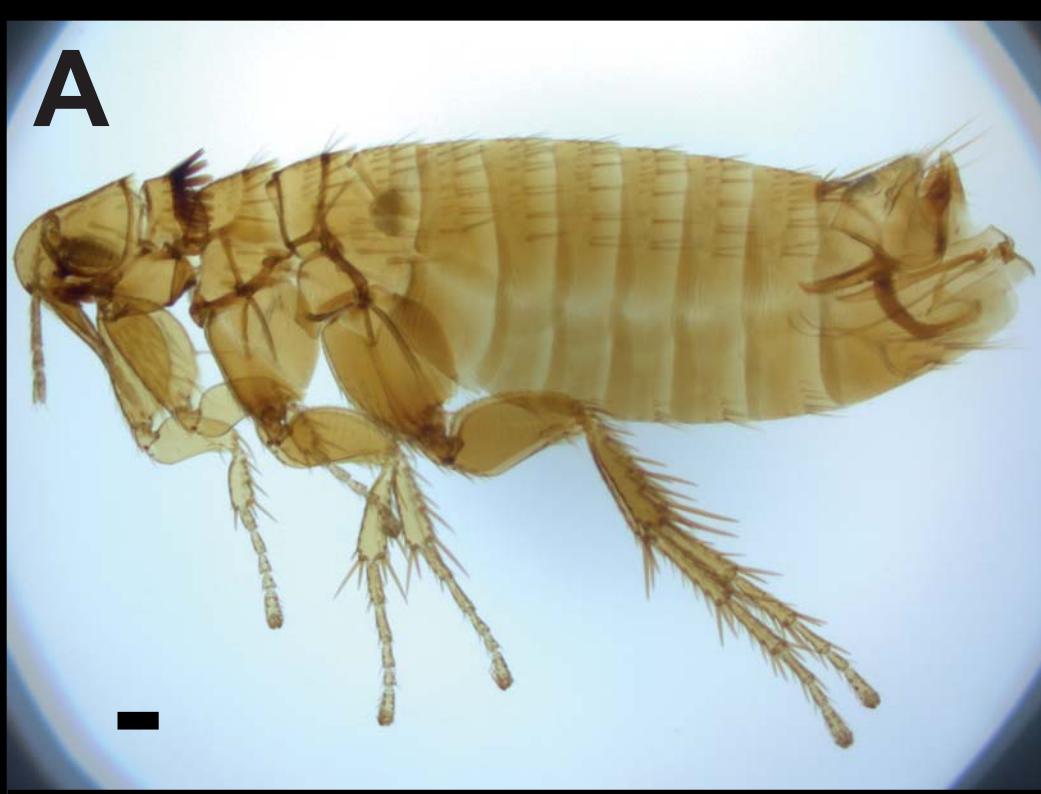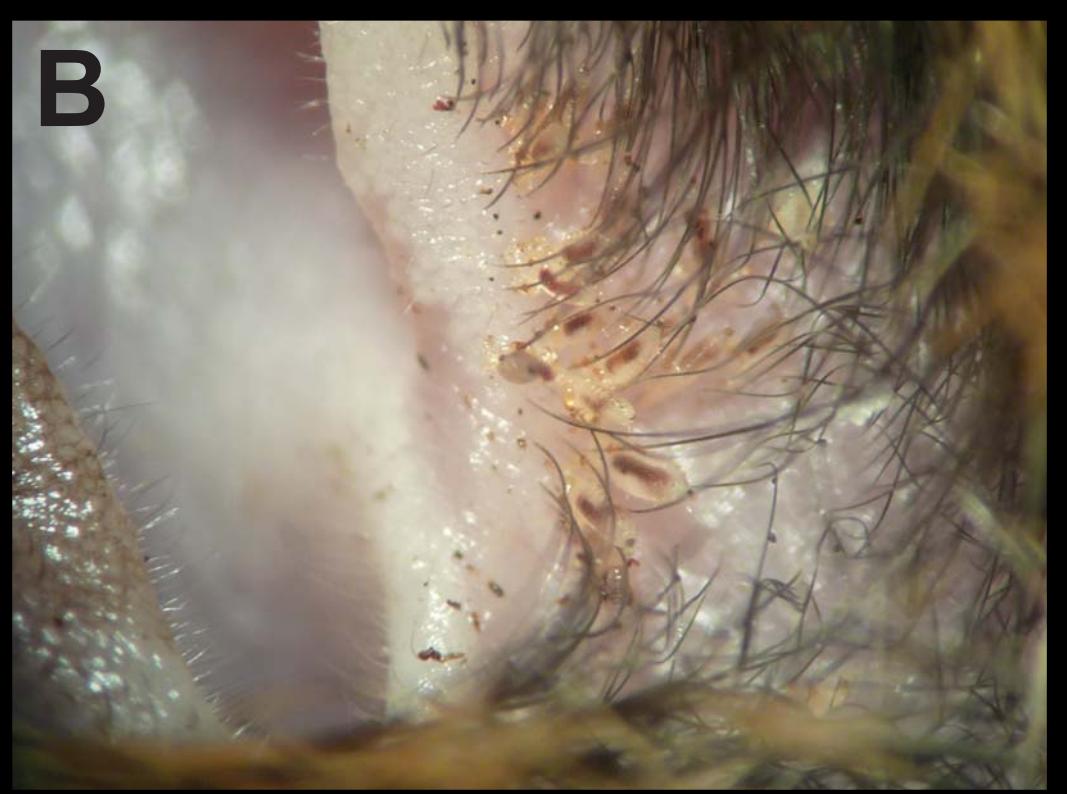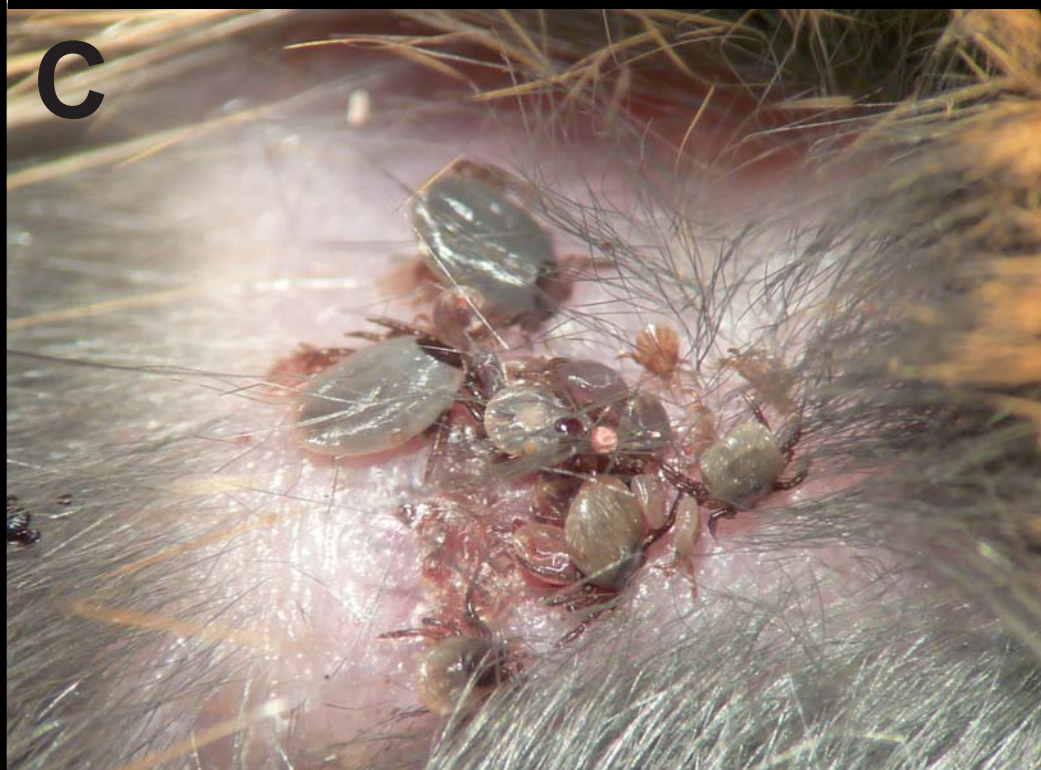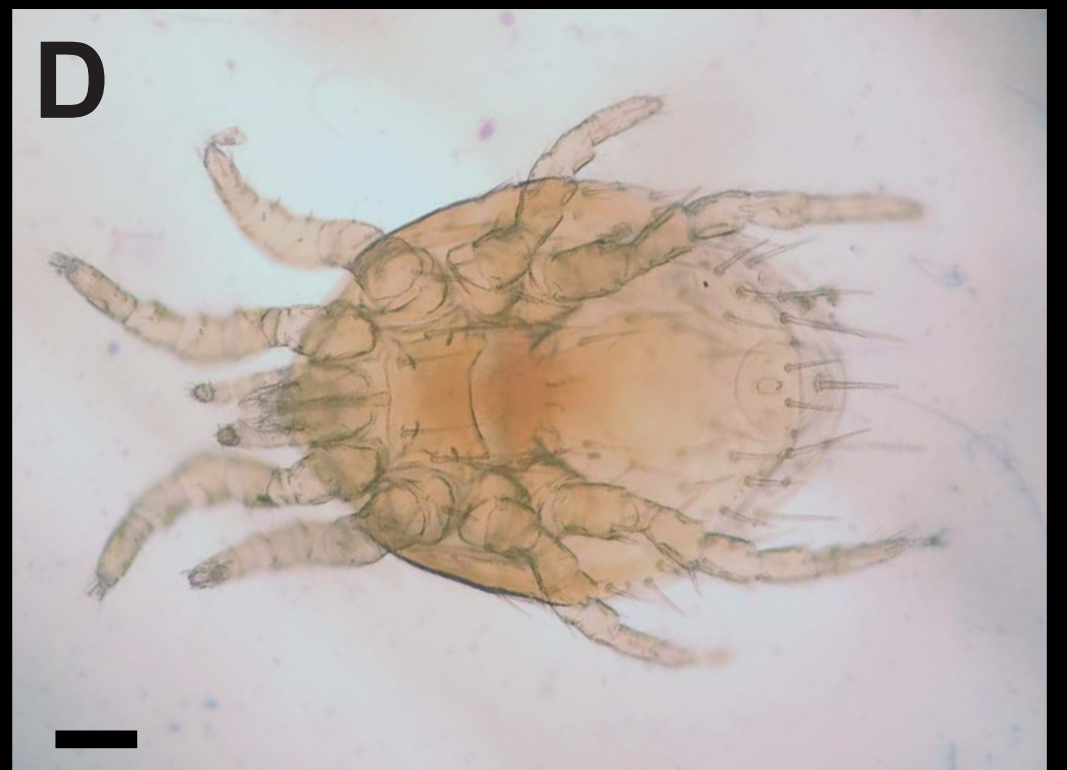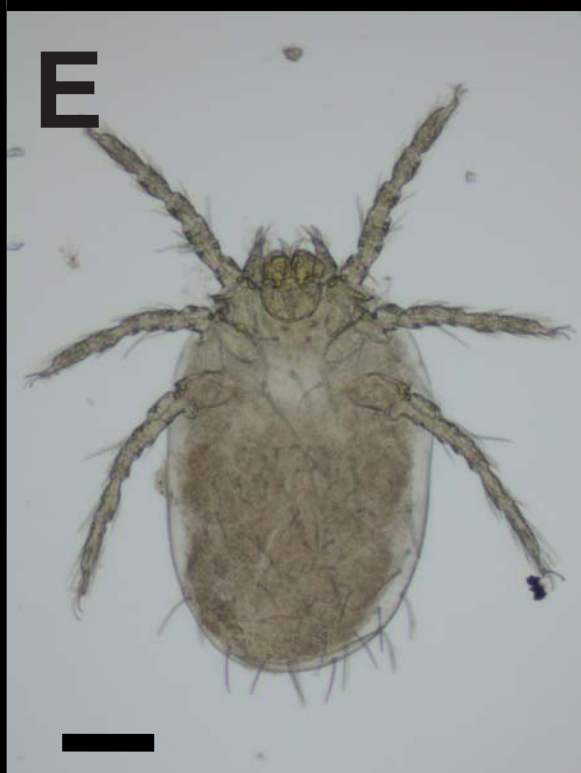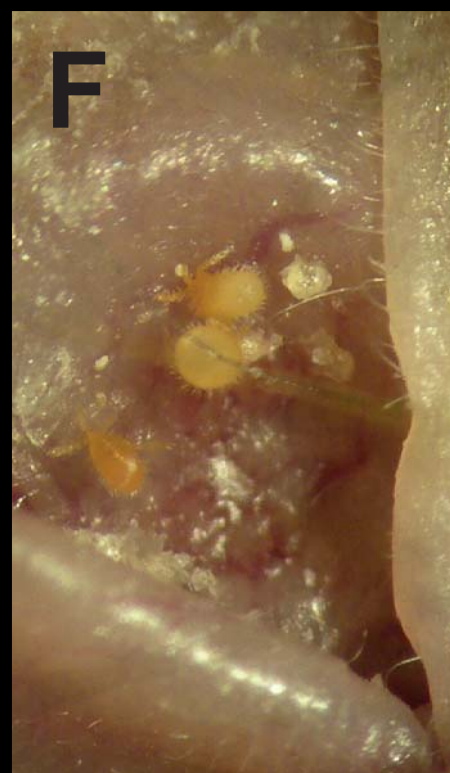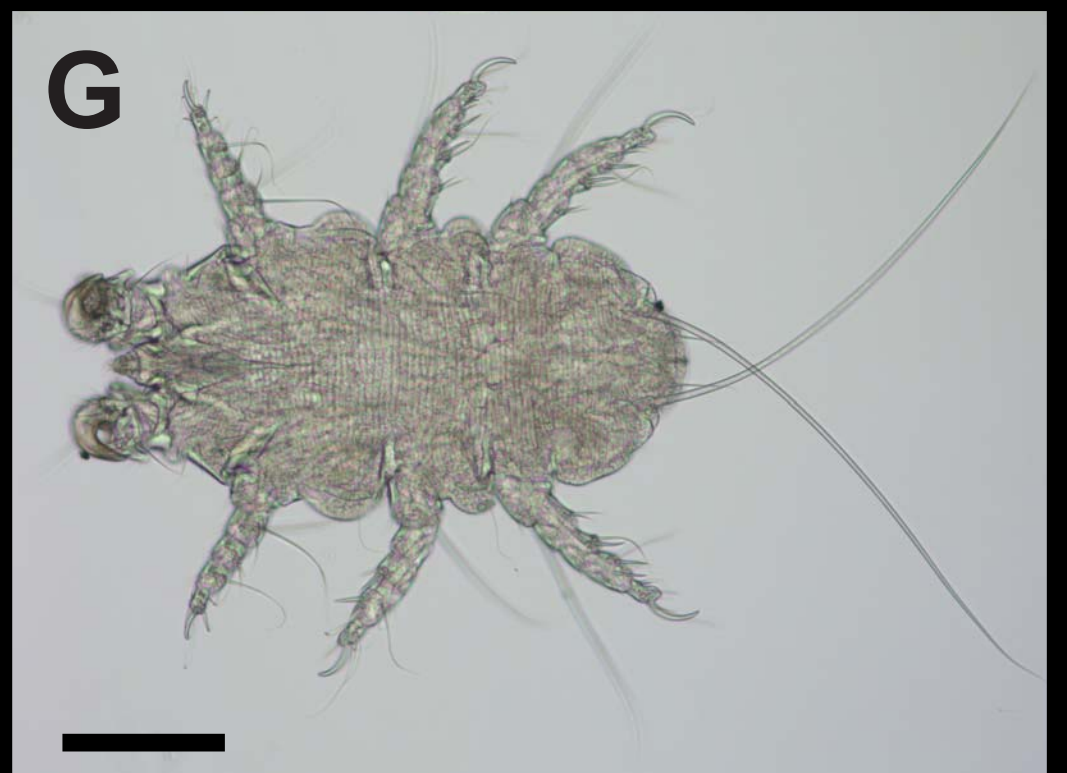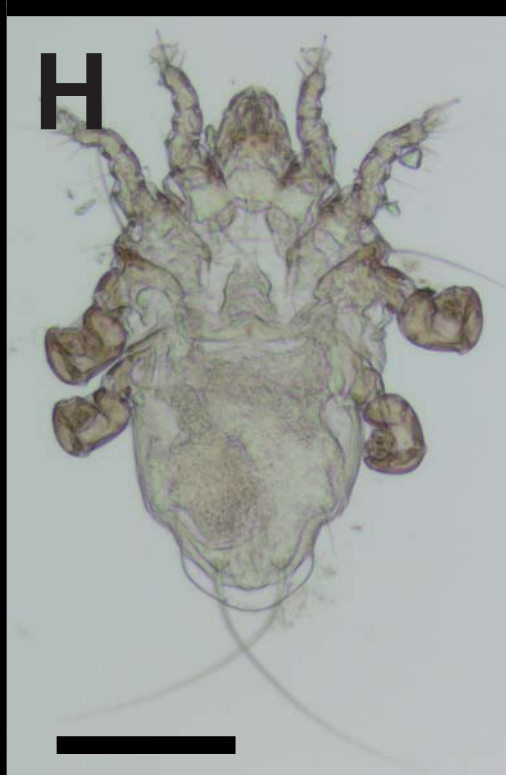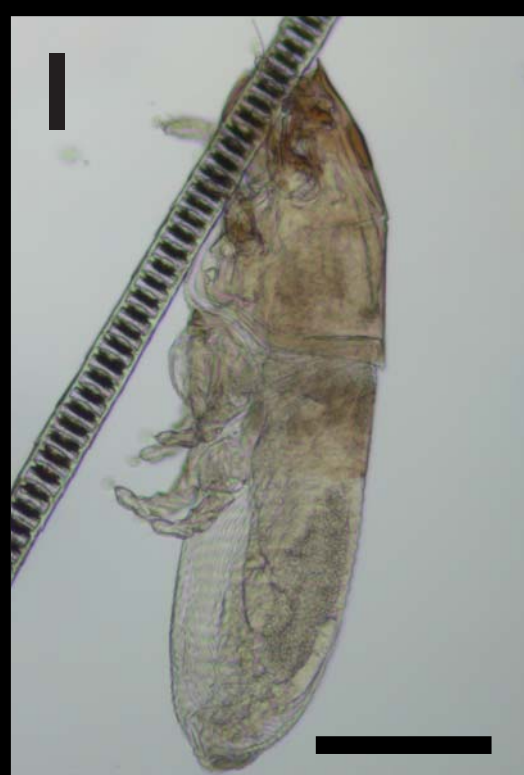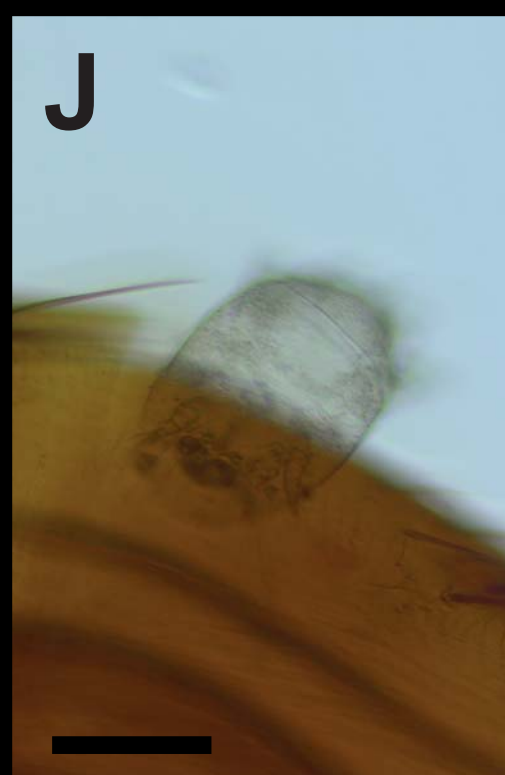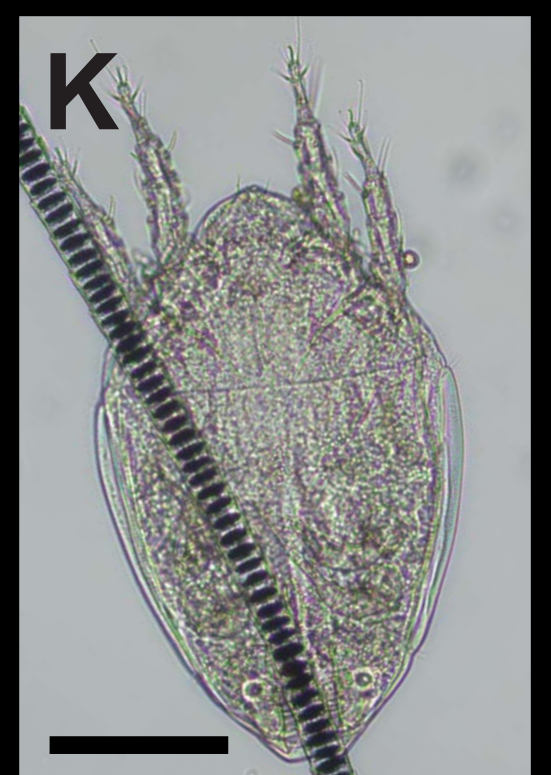

Supplement: S2 Fig — Light micrographs (A, D, E, G-K) and photographs (B, C, F) of diverse rodent-associated arthropod species collected from mice and voles from Berlin. A Ctenophthalmus agyrtes (Siphonaptera) male, B Polyplax serrata (Anoplura) infesting the ear margin of A. agrarius, C Ixodes ricinus (Ixodidae) larvae and nymphs infesting neck of A. flavicollis, D Laelaps hilaris (Laelapidae) female, E Neotrombicula autumnalis (Trombiculidae) larva, F Hirsutiella zachvatkini larvae infesting ear of M. glareolus, G Myobia muris-musculi (Myobiidae) female, H Myocoptes japonensis (Myocoptidae) female, I Afrolistrophorus apodemi (Listrophoridae) female, J phoretic hypopus (deutonymph) of Acarus nidicolous attached to sternal plates of Megabothris turbidus (Siphonaptera), K phoretic hypopus of Glycyphagus hypudaei attached to a hair of M. glareolus, scale bars 0.1 mm, specimens in A and D were cleared in potassium hydroxide. (PDF) [file pone.0199385.s002.pdf]
