# Supplementary material for: Factors associated with diversity, quantity and zoonotic potential of ectoparasites on urban mice and voles
Source: PLoS One. 2018 Jun 25;13(6):e0199385. doi: 10.1371/journal.pone.0199385 (PMC6016914; doi:10.1371/journal.pone.0199385)
Supplement: S3 Fig — Daily means of minute values of saturation deficit calculated from relative humidity and temperature (table 10 in Deutscher Wetterdienst, 1998). Data were measured by a climate station (THIES Clima) at a height of 2 m every day of the year 2011 and were provided by the Institute of Meteorology, Freie Universität Berlin. Line chart (red) is depicted together with an order 4 polynomial trend line (black). (PDF) [file pone.0199385.s003.pdf]

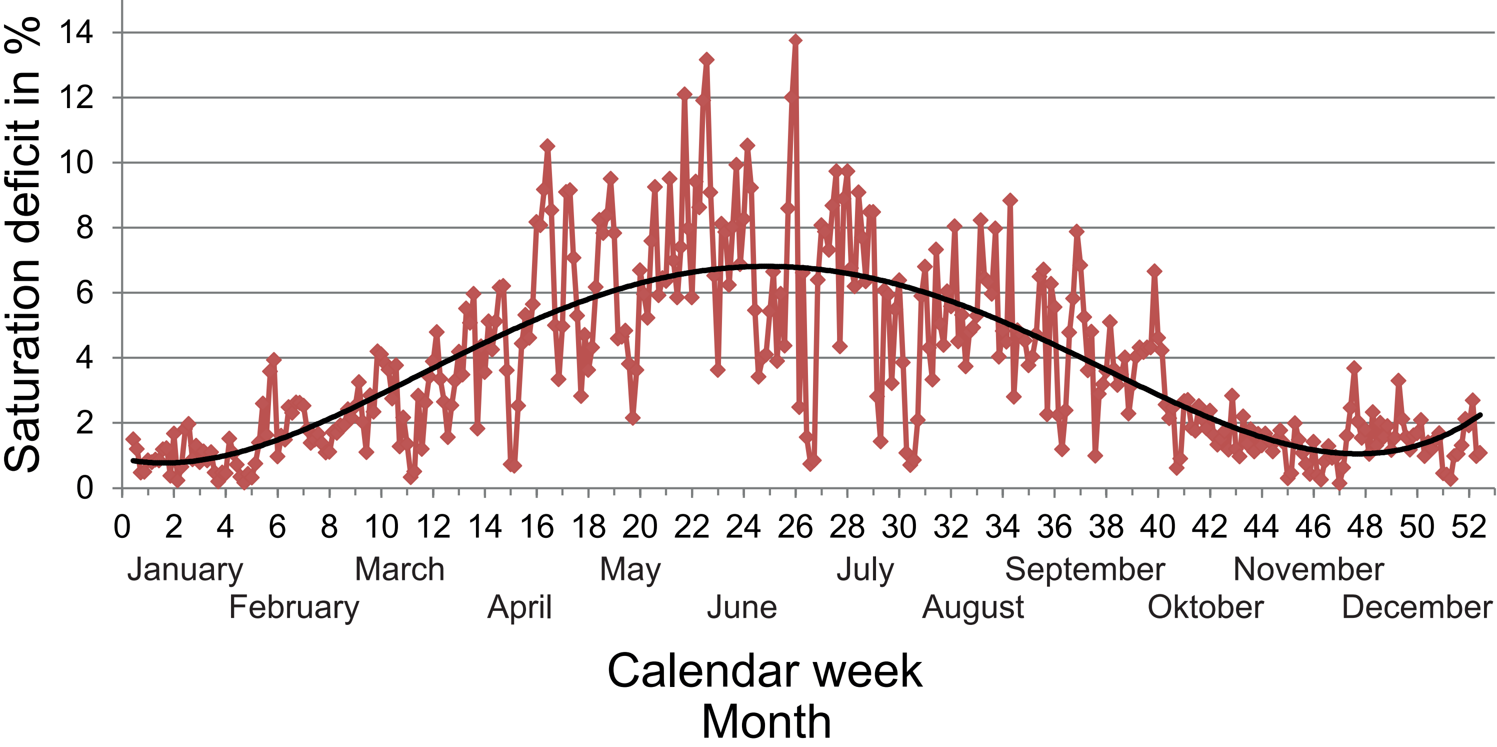

References:

Deutscher Wetterdienst. Aspirations-Psychrometer-Tafeln. 7 ed. Braunschweig: Vieweg Verlag; 1998.
