# Supplementary material for: Factors associated with diversity, quantity and zoonotic potential of ectoparasites on urban mice and voles
Source: PLoS One. 2018 Jun 25;13(6):e0199385. doi: 10.1371/journal.pone.0199385 (PMC6016914; doi:10.1371/journal.pone.0199385)
Supplement: S1 Table — Rodent host species (6 levels) or family (2 levels) and trapping location (4 levels) or location category (2 levels) were used as independent variables. Odds Ratios (OR) for logistic regression (left panel) or Rate Ratios for negative binomial regression (right panel) are shown together with 95% CI. P-values are only shown for one reference level of interest compared to the other levels within the same variable, since correction for multiple testing would drastically increase p-values and hence reduce significances. * p<0.05, ** p<0.01, *** p<0.001, inf: infinite, n.a.: not applicable because of total collinearity between A. sylvaticus and the trapping location Moabit. (DOCX) [file pone.0199385.s004.docx]

| Logistic Regression Analyses | | | | |  | Negative Binomial Regression Analyses | | | | |
| --- | --- | --- | --- | --- | --- | --- | --- | --- | --- | --- |
|  | Odds Ratio | 95%CI | p-Value | |  |  | Rate Ratio | 95%CI | p-Value | |
| **A Prevalence of *N. fasciatus* (Dispersion set to σ^2^=0.936)** | | | | |  | **J Intensity of fleas (Dispersion θ=5.477+/-1.595)** | | | | |
| Intercept | 0.17 | 0.08-0.40 | <0.001 | *** |  | Intercept | 3.7 | 2.88-4.74 | <0.001 | *** |
| Mouse vs. Vole | 0.27 | 0.07-0.99 | 0.047 | * |  | Mouse vs. Vole | 0.57 | 0.42-0.77 | <0.001 | *** |
| Gatow vs. Tegel | 0.23 | 0.03-1.83 | 0.165 |  |  | Gatow vs. Tegel | 1.25 | 0.90-1.73 | 0.184 |  |
| Gatow vs. Steglitz | 2.33 | 0.93-5.82 | 0.070 |  |  | Gatow vs. Steglitz | 0.92 | 0.68-1.23 | 0.560 |  |
| Gatow vs. Moabit | 2.64 | 0.84-8.33 | 0.097 |  |  | Gatow vs. Moabit | 0.7 | 0.41-1.20 | 0.195 |  |
| Tegel vs. Steglitz | 10.09 | 1.39-73.29 |  |  |  | Tegel vs. Steglitz | 0.73 | 0.53-1.02 |  |  |
| Tegel vs. Moabit | 11.45 | 1.40-93.54 |  |  |  | Tegel vs. Moabit | 0.56 | 0.33-0.98 |  |  |
| Steglitz vs. Moabit | 1.13 | 0.46-2.82 |  |  |  | Steglitz vs. Moabit | 0.77 | 0.47-1.27 |  |  |
| **B Prevalence of lice (Dispersion set to σ^2^=0.887)** | | | | |  | **K Intensity of *I. ricinus* larvae (Dispersion θ=1.009+/-0.126)** | | | | |
| Intercept | 2.47 | 1.65-3.69 | <0.001 | *** |  | Intercept | 14.74 | 10.48-20.72 | <0.001 | *** |
| Mouse vs. Vole | 0.25 | 0.11-0.59 | 0.001 | ** |  | Mouse vs. Vole | 0.81 | 0.53-1.23 | 0.320 |  |
| Steglitz vs. Gatow | 0.26 | 0.13-0.51 | <0.001 | *** |  | Gatow vs. Tegel | 0.23 | 0.13-0.43 | <0.001 | *** |
| Steglitz vs. Tegel | 0 | 0-Inf | 0.985 |  |  | Gatow vs. Steglitz | 0.36 | 0.23-0.57 | <0.001 | *** |
| Steglitz vs. Moabit | 0.31 | 0.13-0.72 | 0.006 | ** |  | Gatow vs. Moabit | 0.14 | 0.04-0.42 | <0.001 | *** |
| Gatow vs. Tegel | 0 | 0-Inf |  |  |  | Tegel vs. Steglitz | 1.54 | 0.81-2.92 |  |  |
| Gatow vs. Moabit | 1.21 | 0.48-3.09 |  |  |  | Tegel vs. Moabit | 0.58 | 0.17-1.94 |  |  |
| Tegel vs. Moabit | >999 | 0-Inf |  |  |  | Steglitz vs. Moabit | 0.38 | 0.12-1.16 |  |  |
| **C Prevalence of *I. ricinus* larvae (Dispersion set to σ^2^=1.021)** | | | | |  | **L Intensity of *I. ricinus* larvae (Dispersion θ=1.143+/-0.148)** | | | | |
| Intercept | 5.41 | 2.59-11.31 | <0.001 | *** |  | Intercept | 8.66 | 6.38-11.77 | <0.001 | *** |
| Mouse vs. Vole | 0.89 | 0.40-1.98 | 0.782 |  |  | *M. glareolus* vs. *M. arvalis* | 3.58 | 1.63-7.84 | 0.001 | ** |
| Gatow vs. Tegel | 0.17 | 0.07-0.42 | <0.001 | *** |  | *M. glareolus* vs. *M. agrestis* | 1.21 | 0.30-4.91 | 0.788 |  |
| Gatow vs. Steglitz | 0.13 | 0.06-0.29 | <0.001 | *** |  | *M. glareolus* vs. *A. agrarius* | 1.07 | 0.56-2.05 | 0.831 |  |
| Gatow vs. Moabit | 0.04 | 0.01-0.15 | <0.001 | *** |  | *M. glareolus* vs. *A. flavicollis* | 1.84 | 1.19-2.84 | 0.006 | ** |
| Tegel vs. Steglitz | 0.74 | 0.33-1.68 |  |  |  | *M. glareolus* vs. *A. sylvaticus* | 0.23 | 0.08-0.67 | 0.007 | ** |
| Tegel vs. Moabit | 0.26 | 0.08-0.87 |  |  |  | *M. arvalis* vs. *M. agrestis* | 0.34 | 0.07-1.59 |  |  |
| Steglitz vs. Moabit | 0.35 | 0.12-1.00 |  |  |  | *M. arvalis* vs. *A. agrarius* | 0.30 | 0.13-0.71 |  |  |
| **D Prevalence of parasitic Laelapidae (Dispersion set to σ^2^=1.032)** | | | | |  | *M. arvalis* vs. *A. flavicollis* | 0.52 | 0.24.1.11 |  |  |
| Intercept | 0.45 | 0.25-0.83 | 0.010 | * |  | *M. arvalis* vs. *A. sylvaticus* | n.a. | n.a. |  |  |
| *M. glareolus* vs. *M. arvalis* | 7.57 | 1.39-41.35 | 0.019 | * |  | *M. agrestis* vs. *A. agrarius* | 0.89 | 0.20-3.90 |  |  |
| *M. glareolus* vs. *M. agrestis* | 0 | 0-Inf | 0.989 |  |  | *M. agrestis* vs. *A. flavicollis* | 1.52 | 0.37-6.19 |  |  |
| *M. glareolus* vs. *A. agrarius* | 3.15 | 0.94-10.59 | 0.063 |  |  | *M. agrestis* vs. *A. sylvaticus* | n.a. | n.a. |  |  |
| *M. glareolus* vs. *A. flavicollis* | 5.77 | 2.55-13.02 | <0.001 | *** |  | *A. agrarius* vs. *A. flavicollis* | 1.72 | 0.98-3.00 |  |  |
| *M. glareolus* vs. *A. sylvaticus* | >999 | 0-Inf | 0.991 |  |  | *A. agrarius* vs. *A. sylvaticus* | n.a. | n.a. |  |  |
| *M. arvalis* vs. *M. agrestis* | 0 | 0-Inf |  |  |  | *A. flavicollis* vs. *A. sylvaticus* | n.a. | n.a. |  |  |
| *M. arvalis* vs. *A. agrarius* | 0.42 | 0.07-2.54 |  |  |  | Gatow vs. Tegel | 0.24 | 0.14-0.44 | <0.001 | *** |
| *M. arvalis* vs. *A. flavicollis* | 0.76 | 0.14-4.10 |  |  |  | Gatow vs. Steglitz | 0.43 | 0.26-0.73 | 0.002 | ** |
| *M. arvalis* vs. *A. sylvaticus* | >999 | 0-Inf |  |  |  | Gatow vs. Moabit | n.a. | n.a. | n.a. |  |
| *M. agrestis* vs. *A. agrarius* | >999 | 0-Inf |  |  |  | Tegel vs. Steglitz | 1.76 | 0.88-3.55 |  |  |
| *M. agrestis* vs. *A. flavicollis* | >999 | 0-Inf |  |  |  | Tegel vs. Moabit | n.a. | n.a. |  |  |
| *M. agrestis* vs. *A. sylvaticus* | >999 | 0-Inf |  |  |  | Steglitz vs. Moabit | n.a. | n.a. |  |  |
| *A. agrarius* vs. *A. flavicollis* | 1.83 | 0.65-5.17 |  |  |  | **M Intensity of *I. ricinus* nymphs (Dispersion θ=4.104+/-2.022)** | | | | |
| *A. agrarius* vs. *A. sylvaticus* | >999 | 0-Inf |  |  |  | Intercept | 3.69 | 2.31-5.88 | <0.001 | *** |
| *A. flavicollis* vs. *A. sylvaticus* | >999 | 0-Inf |  |  |  | *A. flavicollis* vs. *M. glareolus* | 0.43 | 0.21-0.87 | 0.020 | * |
| Gatow vs. Tegel | 0.96 | 0.40-2.33 | 0.929 |  |  | *A. flavicollis* vs. *M. arvalis* | 1.08 | 0.37-3.19 | 0.883 |  |
| Gatow vs. Steglitz | 2.45 | 0.87-6.96 | 0.091 |  |  | *A. flavicollis* vs. *A. agrarius* | 0.70 | 0.26-1.85 | 0.471 |  |
| Gatow vs. Moabit | 0 | 0-Inf | 0.992 |  |  | *A. flavicollis* vs. *A. sylvaticus* | 0.34 | 0.11-1.02 | 0.055 |  |
| Tegel vs. Steglitz | 2.55 | 0.76-8.57 |  |  |  | *M. glareolus* vs. *M. arvalis* | 2.53 | 0.82-7.87 |  |  |
| Tegel vs. Moabit | 0 | 0-Inf |  |  |  | *M. glareolus* vs. *A. agrarius* | 1.63 | 0.52-5.13 |  |  |
| Steglitz vs. Moabit | >999 | 0-Inf |  |  |  | *M. glareolus* vs. *A. sylvaticus* | n.a. | n.a. |  |  |
| **E Prevalence of Trombiculidae (Dispersion set to σ^2^=0.490)** | | | | |  | *M. arvalis* vs. *A. agrarius* | 0.65 | 0.16-2.60 |  |  |
| Intercept | 0.04 | 0.01-0.09 | <0.001 | *** |  | *M. arvalis* vs. *A. sylvaticus* | n.a. | n.a. |  |  |
| Vole vs. Mouse | 0.14 | 0.05-0.35 | <0.001 | *** |  | *A. agrarius* vs. *A. sylvaticus* | n.a. | n.a. |  |  |
| Gatow vs. Tegel | 2.40 | 1.10-5.24 | 0.029 | * |  | Gatow vs. Tegel | 0.42 | 0.17-1.01 | 0.052 |  |
| Gatow vs. Steglitz | 0 | 0-Inf | 0.988 |  |  | Gatow vs. Steglitz | 0.43 | 0.18-1.05 | 0.063 |  |
| Gatow vs. Moabit | 0 | 0-Inf | 0.994 |  |  | Gatow vs. Moabit | n.a. | n.a. | n.a. |  |
| Tegel vs. Steglitz | 0 | 0-Inf |  |  |  | Tegel vs. Steglitz | 1.02 | 0.33-3.20 |  |  |
| Tegel vs. Moabit | 0 | 0-Inf |  |  |  | Tegel vs. Moabit | n.a. | n.a. |  |  |
| Steglitz vs. Moabit | 0 | 0-Inf |  |  |  | Steglitz vs. Moabit | n.a. | n.a. |  |  |
| **F Prevalence of *I. ricinus* larvae (Dispersion set to σ^2^=1.025)** | | | | |  | **N Intensity of parasitic Laelapidae (Dispersion θ=1.977+/-0.270)** | | | | |
| Intercept | 4.98 | 2.43-10.21 | <0.001 | *** |  | Intercept | 19.47 | 11.66-32.52 | <0.001 | *** |
| *M. glareolus* vs. *M. arvalis* | 0.72 | 0.15-3.39 | 0.674 |  |  | *M. arvalis* vs. *M. glareolus* | 0.13 | 0.06-0.25 | <0.001 | *** |
| *M. glareolus* vs. *M. agrestis* | >999 | 0-Inf | 0.989 |  |  | *M. arvalis* vs. *A. agrarius* | 0.37 | 0.20-0.67 | 0.001 | ** |
| *M. glareolus* vs. *A. agrarius* | 0.70 | 0.22-2.18 | 0.534 |  |  | *M. arvalis* vs. *A. flavicollis* | 0.63 | 0.36-1.08 | 0.094 |  |
| *M. glareolus* vs. *A. flavicollis* | 1.13 | 0.46-2.74 | 0.792 |  |  | *M. arvalis* vs. *A. sylvaticus* | 0.38 | 0.20-0.69 | 0.002 | ** |
| *M. glareolus* vs. *A. sylvaticus* | >999 | 0-Inf | 0.992 |  |  | *M. glareolus* vs. *A. agrarius* | 2.91 | 1.54-5.47 |  |  |
| *M. arvalis* vs. *M. agrestis* | 0 | 0-Inf |  |  |  | *M. glareolus* vs. *A. flavicollis* | 4.95 | 2.93-8.38 |  |  |
| *M. arvalis* vs. *A. agrarius* | 0.97 | 0.21-4.41 |  |  |  | *M. glareolus* vs. *A. sylvaticus* | n.a. | n.a. |  |  |
| *M. arvalis* vs. *A. flavicollis* | 1.57 | 0.36-6.84 |  |  |  | *A. agrarius* vs. *A. flavicollis* | 1.70 | 1.16-2.51 |  |  |
| *M. arvalis* vs. *A. sylvaticus* | >999 | 0-Inf |  |  |  | *A. agrarius* vs. *A. sylvaticus* | n.a. | n.a. |  |  |
| *M. agrestis* vs. *A. agrarius* | 0 | 0-Inf |  |  |  | *A. flavicollis* vs. *A. sylvaticus* | n.a. | n.a. |  |  |
| *M. agrestis* vs. *A. flavicollis* | 0 | 0-Inf |  |  |  | Gatow vs. Tegel | 0.41 | 0.26-0.66 | <0.001 | *** |
| *M. agrestis* vs. *A. sylvaticus* | 1.25 | 0-Inf |  |  |  | Gatow vs. Steglitz | 0.39 | 0.26-0.57 | <0.001 | *** |
| *A. agrarius* vs. *A. flavicollis* | 1.62 | 0.71-3.70 |  |  |  | Gatow vs. Moabit | n.a. | n.a. | n.a. |  |
| *A. agrarius* vs. *A. sylvaticus* | >999 | 0-Inf |  |  |  | Tegel vs. Steglitz | 0.93 | 0.56-1.55 |  |  |
| *A. flavicollis* vs. *A. sylvaticus* | >999 | 0-Inf |  |  |  | Tegel vs. Moabit | n.a. | n.a. |  |  |
| Gatow vs. Tegel | 0.17 | 0.07-0.41 | <0.001 | *** |  | Steglitz vs. Moabit | n.a. | n.a. |  |  |
| Gatow vs. Steglitz | 0.17 | 0.07-0.44 | <0.001 | *** |  | **O Intensity of Trombiculidae (Dispersion θ=1.754+/-0.713)** | | | | |
| Gatow vs. Moabit | 0 | 0-Inf | 0.991 |  |  | Intercept | 10.12 | 5.06-20.25 | <0.001 | *** |
| Tegel vs. Steglitz | 1.04 | 0.38-2.84 |  |  |  | *M. glareolus* vs. *M. arvalis* | 2.44 | 0.53-11.26 | 0.254 |  |
| Tegel vs. Moabit | 0 | 0-Inf |  |  |  | *M. glareolus* vs. *A. agrarius* | 0.81 | 0.06-10.69 | 0.874 |  |
| Steglitz vs. Moabit | 0 | 0-Inf |  |  |  | *M. glareolus* vs. *A. flavicollis* | 0.95 | 0.29-3.10 | 0.932 |  |
| **G Prevalence of *I. ricinus* nymphs (Dispersion set to σ^2^=1.019)** | | | | |  | *M. arvalis* vs. *M. agrestis* | 0.33 | 0.02-5.41 |  |  |
| Intercept | 0.26 | 0.14-0.49 | <0.001 | *** |  | *M. arvalis* vs. *A. flavicollis* | 0.39 | 0.06-2.49 |  |  |
| *M. glareolus* vs. *M. arvalis* | 1.09 | 0.20-5.98 | 0.925 |  |  | *M. agrestis* vs. *A. flavicollis* | 1.17 | 0.07-18.87 |  |  |
| *M. glareolus* vs. *M. agrestis* | 0 | 0-Inf | 0.989 |  |  | Tegel vs. Gatow | 0.12 | 0.04-0.33 | <0.001 | *** |
| *M. glareolus* vs. *A. agrarius* | 0.89 | 0.23-3.49 | 0.866 |  |  | **P Intensity of Listrophoridae within *Apodemus* mice (Dispersion θ=0.743+/-0.117)** | | | | |
| *M. glareolus* vs. *A. flavicollis* | 1.16 | 0.48-2.79 | 0.744 |  |  | Intercept | 3.67 | 1.33-10.14 | 0.012 | * |
| *M. glareolus* vs. *A. sylvaticus* | 1.55 | 0.30-8.10 | 0.600 |  |  | *A. agrarius* vs. *A. flavicollis* | 1.82 | 0.86-3.83 | 0.116 |  |
| *M. arvalis* vs. *M. agrestis* | 0 | 0-Inf |  |  |  | *A. agrarius* vs. *A. sylvaticus* | 1.31 | 0.61-2.83 | 0.488 |  |
| *M. arvalis* vs. *A. agrarius* | 0.82 | 0.13-5.25 |  |  |  | *A. flavicollis* vs. *A. sylvaticus* | 0.72 | 0.29-1.77 |  |  |
| *M. arvalis* vs. *A. flavicollis* | 1.14 | 0.20-5.59 |  |  |  | Periurban vs. Urban | 2.96 | 1.17-7.48 | 0.022 | * |
| *M. arvalis* vs. *A. sylvaticus* | 1.43 | 0.18-11.24 |  |  |  |  |  |  |  |  |
| *M. agrestis* vs. *A. agrarius* | >999 | 0-Inf |  |  |  |  |  |  |  |  |
| *M. agrestis* vs. *A. flavicollis* | >999 | 0-Inf |  |  |  |  |  |  |  |  |
| *M. agrestis* vs. *A. sylvaticus* | >999 | 0-Inf |  |  |  |  |  |  |  |  |
| *A. agrarius* vs. *A. flavicollis* | 1.30 | 0.40-4.20 |  |  |  |  |  |  |  |  |
| *A. agrarius* vs. *A. sylvaticus* | 1.75 | 0.47-6.51 |  |  |  |  |  |  |  |  |
| *A. flavicollis* vs. *A. sylvaticus* | 1.34 | 0.31-5.85 |  |  |  |  |  |  |  |  |
| Periurban vs. Urban | 0.47 | 0.16-1.39 | 0.178 |  |  |  |  |  |  |  |
| **H Prevalence of Myobiidae (Dispersion set to σ^2^=1.018)** | | | | |  |  |  |  |  |  |
| Intercept | 0.58 | 0.34-0.99 | 0.044 | * |  |  |  |  |  |  |
| *M. glareolus* vs. *M. arvalis* | 0.46 | 0.10-2.10 | 0.316 |  |  |  |  |  |  |  |
| *M. glareolus* vs. *M. agrestis* | >999 | 0-Inf | 0.988 |  |  |  |  |  |  |  |
| *M. glareolus* vs. *A. agrarius* | 0.29 | 0.09-0.87 | 0.027 | * |  |  |  |  |  |  |
| *M. glareolus* vs. *A. flavicollis* | 0.82 | 0.38-1.75 | 0.605 |  |  |  |  |  |  |  |
| *M. glareolus* vs. *A. sylvaticus* | 0.56 | 0.15-2.16 | 0.403 |  |  |  |  |  |  |  |
| *M. arvalis* vs. *M. agrestis* | >999 | 0-Inf |  |  |  |  |  |  |  |  |
| *M. arvalis* vs. *A. agrarius* | 0.62 | 0.14-2.84 |  |  |  |  |  |  |  |  |
| *M. arvalis* vs. *A. flavicollis* | 1.78 | 0.41-7.64 |  |  |  |  |  |  |  |  |
| *M. arvalis* vs. *A. sylvaticus* | 1.22 | 0.22-6.63 |  |  |  |  |  |  |  |  |
| *M. agrestis* vs. *A. agrarius* | 0 | 0-Inf |  |  |  |  |  |  |  |  |
| *M. agrestis* vs. *A. flavicollis* | 0 | 0-Inf |  |  |  |  |  |  |  |  |
| *M. agrestis* vs. *A. sylvaticus* | 0 | 0-Inf |  |  |  |  |  |  |  |  |
| *A. agrarius* vs. *A. flavicollis* | 2.87 | 1.16-7.05 |  |  |  |  |  |  |  |  |
| *A. agrarius* vs. *A. sylvaticus* | 1.98 | 0.75-5.19 |  |  |  |  |  |  |  |  |
| *A. flavicollis* vs. *A. sylvaticus* | 0.69 | 0.22-2.21 |  |  |  |  |  |  |  |  |
| Periurban vs. Urban | 6.53 | 2.65-16.12 | <0.001 | *** |  |  |  |  |  |  |
| **I Prevalence of Listrophoridae within *Apodemus* mice (Dispersion set to σ^2^=1.017)** | | | | |  |  |  |  |  |  |
| Intercept | 0.17 | 0.06-0.46 | <0.001 | *** |  |  |  |  |  |  |
| *A. agrarius* vs. *A. flavicollis* | 1.56 | 0.67-3.64 | 0.306 |  |  |  |  |  |  |  |
| *A. agrarius* vs. *A. sylvaticus* | 1.41 | 0.56-3.53 | 0.463 |  |  |  |  |  |  |  |
| *A. flavicollis* vs. *A. sylvaticus* | 0.90 | 0.31-2.66 |  |  |  |  |  |  |  |  |
| Periurban vs. Urban | 4.64 | 1.81-11.86 | 0.001 | ** |  |  |  |  |  |  |
