# Supplementary material for: Factors associated with diversity, quantity and zoonotic potential of ectoparasites on urban mice and voles
Source: PLoS One. 2018 Jun 25;13(6):e0199385. doi: 10.1371/journal.pone.0199385 (PMC6016914; doi:10.1371/journal.pone.0199385)
Supplement: S2 Table — Total number and sex ratio (male: female) of parasites as well as number of infected rodents, prevalence and mean intensity for six rodent species are shown for every arthropod species. The number of examined rodents and the number of male/female are given below the species name. The last column shows values for the sum of all rodent species. Capital letters next to the families indicate higher arthropod taxa: Si Siphonaptera (fleas), Ph Phthiraptera (lice), Ix Ixodida (ticks), Ga Gamasina (Mesostigmata), Pr Prostigmata, As Astigmata. n: Number of arthropods. Hyphens indicate absence of arthropods. No: Number of infested rodents. P [%]: Prevalence in %. mI [n]: mean intensity = mean number of parasites on infected rodents. max: highest arthropod intensity. n.d.: no adults observed or not determined. (DOCX) [file pone.0199385.s005.docx]

|  | Arthropod | | | | | | | | |  | Rodent host | | | | | | | | | | | | | | | | | | | | | | | | | | | |
| --- | --- | --- | --- | --- | --- | --- | --- | --- | --- | --- | --- | --- | --- | --- | --- | --- | --- | --- | --- | --- | --- | --- | --- | --- | --- | --- | --- | --- | --- | --- | --- | --- | --- | --- | --- | --- | --- | --- |
|  |  | | |  | |  |  |  |  |  | *M. glareolus* | | |  | *M. arvalis* | | |  | *M. agrestis* | | |  | *A. agrarius* | | |  | *A. flavicollis* | | |  | *A. sylvaticus* | | |  | All Species | | | |
|  |  | | |  | |  |  |  |  |  | 29/29 = 59^a^ | | |  | 4/7 = 11 | | |  | 0/2 = 2 | | |  | 44/33 = 77 | | |  | 41/41 = 82 | | |  | 14/11 = 25 | | |  | 132/123 = 256^a^ | | | |
|  |  | | | Species | | | n | Sex ratio | |  | No | P [%] | mI [n] |  | No | P [%] | mI [n] |  | No | P [%] | mI [n] |  | No | P [%] | mI [n] |  | No | P [%] | mI [n] |  | No | P [%] | mI [n] |  | No | P [%] | mI [n] | max |
| **Parasitic arthropods** | | | | |  | |  |  |  |  |  |  |  |  |  |  |  |  |  |  |  |  |  |  |  |  |  |  |  |  |  |  |  |  |  |  |  |  |
|  | **Si** | | Ctenophthalmidae | | *Ctenophthalmus agyrtes* | | 369 | 1 : | 1.31 |  | 27 | 46 | 1.8 |  | 6 | 55 | 2.3 |  | 1 | 50 | 1.0 |  | 38 | 49 | 2.8 |  | 52 | 63 | 3.7 |  | 4 | 16 | 2.2 |  | 128 | 50.0 | 2.9 | 14 |
|  |  | |  | | *Ctenopthalmus assimilis* | | 7 | 1 : | 1.3 |  | 3 | 5 | 1.0 |  | 1 | 9 | 3.0 |  | 1 | 50 | 1.0 |  | - | | |  | - | | |  | - | | |  | 5 | 2.0 | 1.4 | 3 |
|  |  | |  | | *Ctenophthalmus congerer* | | 9 | 1 : | 0.3 |  | 6 | 10 | 1.3 |  | 1 | 9 | 1.0 |  | - | | |  | - | | |  | - | | |  | - | | |  | 7 | 2.7 | 1.3 | 2 |
|  |  | |  | | *Rhadinopsylla pentacantha* | | 11 | 1 : | 1.2 |  | 4 | 7 | 1.8 |  | 1 | 9 | 1.0 |  | - | | |  | 1 | 1 | 2.0 |  | 1 | 1 | 1.0 |  | - | | |  | 7 | 2.7 | 1.6 | 3 |
|  | **Si** | | Hystrichopsyllidae | | *Hystrichopsylla orientalis* | | 5 | 1 : | 4.0 |  | 2 | 3 | 1.0 |  | 2 | 18 | 1.0 |  | - | | |  | 1 | 1 | 1.0 |  | - | | |  | - | | |  | 5 | 2.0 | 1.0 | 1 |
|  |  | |  | | *Typhloceras poppei* | | 15 | 1 : | 0.7 |  | - | | |  | - | | |  | - | | |  | 9 | 12 | 1.3 |  | - | | |  | 1 | 4 | 3.0 |  | 10 | 3.9 | 1.5 | 3 |
|  | **Si** | | Ceratophyllidae | | *Peromyscopsylla sylvatica* | | 3 | 1 : | 2.0 |  | 2 | 3 | 1.0 |  | 1 | 9 | 1.0 |  | - | | |  | - | | |  | - | | |  | - | | |  | 3 | 1.2 | 1.0 | 1 |
|  |  | |  | | *Nosopsyllus fasciatus* | | 68 | 1 : | 0.9 |  | 1 | 2 | 1.0 |  | 2 | 18 | 1.0 |  | - | | |  | 13 | 17 | 1.3 |  | 22 | 27 | 1.6 |  | 8 | 32 | 1.5 |  | 46 | 18.0 | 1.5 | 8 |
|  |  | |  | | *Megabothris turbidus* | | 15 | 1 : | 4.0 |  | 8 | 14 | 1.5 |  | - | | |  | - | | |  | - | | |  | 3 | 4 | 1.0 |  | - | | |  | 11 | 4.3 | 1.4 | 2 |
|  |  | |  | | *Monopsyllus sciurorum* | | 2 | 1 : | 1.0 |  | - | | |  | 1 | 9 | 1.0 |  | - | | |  | 1 | 1 | 1.0 |  | - | | |  | - | | |  | 2 | 0.8 | 1.0 | 1 |
|  | **Ph** | | Polyplacidae | | *Polyplax serrata* | | 373 | 1 : | 2.2 |  | - | | |  | - | | |  | - | | |  | 63 | 82 | 4.7 |  | 20 | 24 | 2.9 |  | 10 | 40 | 1.9 |  | 93 | 36.3 | 4.0 | 34 |
|  | **Ph** | | Hoplopleuridae | | *Hoplopleura affinis* | | 59 | 1 : | 2.9 |  | - | | |  | - | | |  | - | | |  | 3 | 4 | 20.0 |  | - | | |  | - | | |  | 3 | 1.2 | 19.7 | 44 |
|  |  | |  | | *Hoplopleura acanthopus* | | 15 | 1 : | 0.8 |  | - | | |  | 3 | 27 | 5.0 |  | - | | |  | - | | |  | - | | |  | - | | |  | 3 | 1.2 | 5.0 | 3 |
|  |  | |  | | *Hoplopleura edentula* | | 16 | 1 : | 1.6 |  | 6 | 10 | 2.7 |  | - | | |  | - | | |  | - | | |  | - | | |  | - | | |  | 6 | 2.3 | 2.7 | 6 |
|  | **Ix** | | Ixodidae | | *Ixodes ricinus* total | | 1359 | n.d. | |  | 44 | 75 | 7.9 |  | 8 | 73 | 27.0 |  | 2 | 100 | 10.5 |  | 31 | 40 | 5.4 |  | 53 | 65 | 11.2 |  | 6 | 24 | 2.5 |  | 144 | 56.3 | 9.4 | 108 |
|  |  | |  | | larvae | | 1277 |  | |  | 43 | 73 | 7.7 |  | 7 | 64 | 29.7 |  | 2 | 100 | 10.5 |  | 31 | 40 | 5.1 |  | 52 | 63 | 10.6 |  | 5 | 20 | 2.0 |  | 140 | 54.7 | 9.1 | 101 |
|  |  | |  | | nymphs | | 82 |  | |  | 12 | 20 | 1.3 |  | 2 | 18 | 4.0 |  | - | | |  | 8 | 10 | 1.2 |  | 16 | 20 | 2.7 |  | 4 | 16 | 1.2 |  | 42 | 16.4 | 2.0 | 17 |
|  |  | |  | | *Ixodes trianguliceps* total | | 9 | n.d. | |  | 4 | 7 | 1.8 |  | - | | |  | - | | |  | - | | |  | 2 | 2 | 1.0 |  | - | | |  | 6 | 2.3 | 1.5 | 3 |
|  |  | |  | | larvae | | 6 |  | |  | 3 | 5 | 1.7 |  | - | | |  | - | | |  | - | | |  | 1 | 1 | 1.0 |  | - | | |  | 4 | 1.6 | 1.5 | 3 |
|  |  | |  | | nymphs | | 3 |  | |  | 1 | 2 | 2.0 |  | - | | |  | - | | |  | - | | |  | 1 | 1 | 1.0 |  | - | | |  | 2 | 0.8 | 1.5 | 2 |
|  |  | |  | | *Dermacentor* *reticulatus* (nymphs) | | 2 | n.d. | |  | 1 | 2 | 2.0 |  | - | | |  | - | | |  | - | | |  | - | | |  | - | | |  | 1 | 0.4 | 2.0 | 2 |
|  | **Ga** | | Laelapidae | | *Laelaps agilis* | | 405 | 1 : | 7.7 |  | 1 | 2 | 1.0 |  | - | | |  | - | | |  | - | | |  | 46 | 56 | 6.7 |  | 17 | 68 | 5.6 |  | 64 | 25.0 | 6.3 | 56 |
|  |  | |  | | *Laelaps jettmari (L. pavlovskyi)* | | 48 | 1 : | 11.0 |  | - | | |  | - | | |  | - | | |  | 22 | 29 | 2.1 |  | 1 | 1 | 1.0 |  | - | | |  | 23 | 9.0 | 2.1 | 8 |
|  |  | |  | | *Laelaps hilaris* | | 87 | 1 : | 9.7 |  | - | | |  | 7 | 64 | 12.3 |  | - | | |  | 1 | 1 | 1.0 |  | - | | |  | - | | |  | 8 | 3.1 | 10.9 | 35 |
|  |  | |  | | *Hyperlaelaps microti* | | 21 | 1 : | 3.0 |  | - | | |  | 5 | 46 | 4.2 |  | - | | |  | - | | |  | - | | |  | - | | |  | 5 | 2.0 | 4.2 | 7 |
|  |  | |  | | *Androlaelaps fahrenholzi* | | 3 | 1 : | >3.0 |  | 1 | 2 | 1.0 |  | 1 | 9 | 1.0 |  | - | | |  | - | | |  | 1 | 1 | 1.0 |  | - | | |  | 3 | 1.2 | 1.0 | 1 |
|  |  | |  | | *Haemogamasus nidi* | | 89 | 1 : | 12.3 |  | 5 | 9 | 1.4 |  | 4 | 36 | 1.5 |  | - | | |  | 5 | 7 | 1.2 |  | 18 | 22 | 2.7 |  | 6 | 24 | 3.7 |  | 38 | 14.8 | 2.3 | 16 |
|  |  | |  | | *Haemogamasus hirsutus* | | 1 | n.d. | |  | - | | |  | - | | |  | - | | |  | - | | |  | 1 | 1 | 1.0 |  | - | | |  | 1 | 0.4 | 1.0 | 1 |
|  |  | |  | | *Haemogamasus hirsutosimilis* | | 3 | n.d. | |  | - | | |  | - | | |  | - | | |  | - | | |  | 1 | 1 | 3.0 |  | - | | |  | 1 | 0.4 | 3.0 | 3 |
|  |  | |  | | *Eulaelaps stabularis* | | 49 | 1 : | 48.0 |  | 2 | 3 | 1.0 |  | 1 | 9 | 1.0 |  | - | | |  | 5 | 7 | 2.2 |  | 9 | 11 | 1.9 |  | 8 | 32 | 2.2 |  | 25 | 9.8 | 2.0 | 7 |
|  |  | |  | | *Hirstionyssus* (*Echinonyssus*) *sunci* | | 241 | 1 : | 116.5 |  | 3 | 5 | 1.3 |  | 2 | 18 | 3.0 |  | - | | |  | 47 | 61 | 2.2 |  | 25 | 31 | 4.1 |  | 12 | 48 | 2.2 |  | 89 | 34.8 | 2.7 | 27 |
|  |  | |  | | *Hirstionyssus* (*Echinonyssus*) *isabellinus* | | 22 | 1 : | 20.0 |  | 9 | 15 | 2.4 |  | - | | |  | - | | |  | - | | |  | - | | |  | - | | |  | 9 | 3.5 | 2.4 | 4 |
|  |  | |  | | *Hirstionyssus* (*Echinonyssus*) *soricis* | | 1 |  |  |  | 1 | 2 | 1.0 |  | - | | |  | - | | |  | - | | |  | - | | |  | - | | |  | 1 | 0.4 | 1.0 | 1 |
|  |  | |  | | *Myonyssus gigas* | | 17 | 1 : | 14.0 |  | - | | |  | - | | |  | - | | |  | - | | |  | 5 | 6 | 3.4 |  | - | | |  | 5 | 2.0 | 3.4 | 9 |
|  | **Pr** | | Myobiidae | | *Myobia muris-musculi* | | 102 | 1 : | 4.8 |  | - | | |  | - | | |  | - | | |  | - | | |  | 28 | 34 | 2.5 |  | 12 | 48 | 2.7 |  | 40 | 15.6 | 2.6 | 10 |
|  |  | |  | | *Myobia multivaga* | | 95 | 1 : | 7.6 |  | - | | |  | - | | |  | - | | |  | - | | |  | 14 | 17 | 3.7 |  | 11 | 44 | 3.9 |  | 25 | 9.8 | 3.8 | 15 |
|  |  | |  | | *Myobia agraria* | | 100 | 1 : | 7.4 |  | - | | |  | - | | |  | - | | |  | 38 | 49 | 2.6 |  | - | | |  | - | | |  | 38 | 14.8 | 2.6 | 12 |
|  |  | |  | | *Radfordia lemnia* | | 31 | 1 : | 0.5 |  | - | | |  | 4 | 36 | 2.2 |  | 2 | 100 | 11.0 |  | - | | |  | - | | |  | - | | |  | 6 | 2.3 | 5.2 | 14 |
|  |  | |  | | *Radfordia clethrionomys* | | 88 | 1 : | 1.9 |  | 22 | 37 | 4.0 |  | - | | |  | - | | |  | - | | |  | - | | |  | - | | |  | 22 | 8.6 | 4.0 | 17 |
|  |  | |  | | *Radfordia lancearia* | | 26 | 1 : | 5.0 |  | - | | |  | - | | |  | - | | |  | - | | |  | 10 | 12 | 2.0 |  | 4 | 16 | 1.5 |  | 14 | 5.5 | 1.9 | 5 |
|  | **Pr** | | Trombiculidae | | *Neotrombicula autumnalis* | | 22 | n.d. | |  | 8 | 14 | 1.1 |  | 2 | 18 | 3.0 |  | 1 | 2 | 1.0 |  | - | | |  | 2 | 2 | 3.0 |  | - | | |  | 13 | 5.1 | 1.7 | 5 |
|  |  | |  | | *Hirsutiella zachvatkini* | | 66 | n.d. | |  | 5 | 9 | 11.0 |  | - | | |  | - | | |  | - | | |  | 2 | 2 | 6.0 |  | - | | |  | 7 | 2.7 | 9.4 | 28 |
|  | **Pr** | | Psorergatidae^d^ | | Psorergates spec. | | ≥ 10 | n.d. | |  | - | | |  | - | | |  | - | | |  | - | | |  | 1 |  |  |  | - | | |  | 1 |  |  |  |
|  | **Pr** | | Ereynetidae^d^ | | *Paraspeleognathopsis bakeri* | | ≥ 2 | n.d. | |  | 1 |  |  |  | 1 |  |  |  | - | | |  | - | | |  | - | | |  | - | | |  | 2 |  |  |  |
|  | **Pr** | | Demodicidae^d^ | | *Demodex* spec. | | ≥ 1 | n.d. | |  | - | | |  | - | | |  | - | | |  | - | | |  | 1 |  |  |  | - | | |  | 1 |  |  |  |
|  | **As** | | Myocoptidae^b^ | | *Myocoptes japonensis* | | 160 | 1 : | 2.8 |  | 22 | 37 | ≥5.7 |  | 6 | 55 | ≥5.8 |  | - | | |  | - | | |  | - | | |  | - | | |  | 28 | 10.9 | ≥5.7 | ≥24 |
|  |  | |  | | *Trichoecius tenax* | | 132 | 1 : | 4.8 |  | 25 | 42 | ≥4.4 |  | 5 | 36 | ≥4.8 |  | 1 | 50 | ≥2.0 |  | - | | |  | - | | |  | - | | |  | 30 | 11.7 | ≥4.4 | ≥20 |
|  |  | |  | | *Trichoecius widawaensis* | | 3 | 1 : | 2.0 |  | - | | |  | - | | |  | - | | |  | 3 | 4 | ≥1.0 |  | - | | |  | - | | |  | 3 | 1.2 | ≥1 | 1 |
|  |  | |  | | *Criniscansor* spec. | | 4 | n.d. | |  | - | | |  | - | | |  | - | | |  | 3 | 4 | ≥1.0 |  | 1 | 1 | ≥1.0 |  | - | | |  | 4 | 1.6 | ≥1 | 1 |
|  | **As** | | Listrophoridae^b^ | | *Listrophorus brevipes* | | 169 | n.d. | |  | 9 | 15 | ≥16.0 |  | - | | |  | 2 | 100 | ≥13.0 |  | - | | |  | - | | |  | - | | |  | 11 | 4.3 | ≥15.4 | ≥100 |
|  |  | |  | | *Afrolistrophorus apodemi* | | 888 | n.d. | |  | - | | |  | - | | |  | - | | |  | 32 | 42 | ≥11.0 |  | 26 | 32 | ≥14.0 |  | 13 | 52 | ≥14.0 |  | 71 | 27.7 | ≥12.5 | ≥146 |
|  | **As** | | Gastronyssidae^d^ | | *Yunkeracarus apodemi* | | ≥ 2 | n.d. | |  | - | | |  | - | | |  | - | | |  | 1 |  |  |  | - | | |  | - | | |  | 1 |  |  |  |
|  | **As** | | Glycyphagidae^d^ | | *Lophioglyphus liciosus* (*Apodemopus apodemi*) | | ≥ 1 | n.d. | |  | - | | |  | - | | |  | - | | |  | - | | |  | - | | |  | 1 |  |  |  | 1 |  |  |  |
| **Phoretic arthropods** | | | | |  | |  |  |  |  | - | | |  | - | | |  |  | | |  |  | | |  |  | | |  |  | | |  |  |  |  |  |
|  | | **P** | | Pygmephoridae | *Pygmephorus forcipatus* | | 2 | 1 : | > 2 |  | - | | |  | 1 | 9 | 2.0 |  | - | | |  | - | | |  | - | | |  | - | | |  | 1 | 0.4 | 2.0 | 2 |
|  | | **As** | | Glycyphagidae | *Xenoryctes krameri* (hypopi) | | 82 | n.d. | |  | 7 | 12 | 2.7 |  | 2 | 18 | 4.0 |  | 1 | 50 | 1.0 |  | 17 | 22 | 2.7 |  | 3 | 4 | 2.7 |  | - | | |  | 30 | 11.7 | 2.7 | 10 |
|  | |  | |  | *Glycyphagus hypudaei* (hypopi) | | 78 | n.d. | |  | 3 | 5 | 1.0 |  | - | | |  | 1 | 50 | 1.0 |  | 2 | 3 | 2.5 |  | 3 | 4 | 23.0 |  | - | | |  | 9 | 3.5 | 8.7 | 56 |
|  | | **As** | | Acaridae | *Acarus nidicolous* (hypopi)^c^ | | 16 | n.d. | |  | 4 | 7 | 2.0 |  | - | | |  |  | | |  | - | | |  | 5 | 6 | 1.6 |  | - | | |  | 9 | 3.5 | 1.8 | 4 |
| **Nidicolous arthropods** | | | | |  | |  |  |  |  | - | | |  | - | | |  |  | | |  |  |  |  |  |  | | |  |  | | |  |  |  |  |  |
|  | | **Ga** | | Laelapidae | *Hypoaspis sardoa* | | 3 | 1 : | > 3 |  | - | | |  | - | | |  | - | | |  | 1 | 1 | 1.0 |  | 2 | 2 | 1.0 |  | - | | |  | 3 | 1.2 | 1.0 | 1 |
|  | | **Ga** | | Ologamasidae | *Euryparasitus emarginatus* | | 2 | n.d. | |  | - | | |  | - | | |  | - | | |  | - | | |  | 2 | 2 | 1.0 |  | - | | |  | 2 | 0.8 | 1.0 | 1 |
|  | |  | |  | *Cyrtolaelaps mucronatus* | | 3 | n.d. | |  | - | | |  | 1 | 9 | 1.0 |  | 1 | 50 | 1.0 |  | 1 | 1 | 1.0 |  | - | | |  | - | | |  | 3 | 1.2 | 1.0 | 1 |
|  | | **Ga** | | Parasitidae | Parasitinae spec. | | 2 | n.d. | |  | - | | |  | - | | |  | - | | |  | - | | |  | - | | |  | 2 | 8 | 1.0 |  | 2 | 0.8 | 1.0 | 1 |
|  | |  | |  | *Pergamasus* spec. | | 1 | n.d. | |  | - | | |  | - | | |  | - | | |  | - | | |  | 1 | 1 | 1.0 |  | - | | |  | 1 | 0.4 | 1.0 | 1 |
|  | |  | |  | *Eugamasus* spec. | | 1 | n.d. | |  | - | | |  | - | | |  | - | | |  | - | | |  | 1 | 1 | 1.0 |  | - | | |  | 1 | 0.4 | 1.0 | 1 |
|  | | **Pr** | | Cheyletidae | *Eucheyletia flabellifera* | | 6 | 1 : | >4 |  | - | | |  | 2 | 9 | 3.0 |  | - | | |  | - | | |  | - | | |  | - | | |  | 2 | 0.8 | 3.0 | 5 |
|  | | **As** | | Glycyphagidae | *Glycyphagus domesticus* | | 4 | 1 : | <0.5 |  | - | | |  | 1 | 9 | 1.0 |  | - | | |  | 1 | 1 | 1.0 |  | 1 | 1 | 1.0 |  | 1 | 4 | 1.0 |  | 4 | 1.6 | 1.0 | 1 |
|  | | **As** | | Acaridae | *Tyrophagus dimidiatus* | | 2 | 1 : | >2 |  | - | | |  | - | | |  | - | | |  | 1 | 1 | 1.0 |  | - | | |  | 1 | 4 | 1.0 |  | 2 | 0.8 | 1.0 | 1 |
|  | |  | |  | Acaridae spec. | | 1 | n.d. | |  | - | | |  | 1 | 9 | 1.0 |  | - | | |  | - | | |  | - | | |  | - | | |  | 1 | 0.4 | 1.0 | 1 |

^a^ Sex of one bank vole was not determined

^b^ Because of small body size of Myocoptidae and Listrophoridae, not all specimens were sampled when high intensities occurred. Values should be treated as minimum numbers. Sex ratio of Listrophoridae was not determined

^c^ The mite was not directly phoretic on rodents but on fleas

^d^ Incidental findings of skin-inhabiting or nasal mites without quantification
