# Supplementary material for: Factors associated with diversity, quantity and zoonotic potential of ectoparasites on urban mice and voles
Source: PLoS One. 2018 Jun 25;13(6):e0199385. doi: 10.1371/journal.pone.0199385 (PMC6016914; doi:10.1371/journal.pone.0199385)
Supplement: S3 Table — All variables have a variance inflation factor below a threshold of 5 and therefore can be considered to have low collinearity with the other variables. (DOCX) [file pone.0199385.s006.docx]

| Variable | Variance inflation factor |
| --- | --- |
| Age | 1.156 |
| Body condition | 1.055 |
| Fleas count | 1.143 |
| Lice count | 1.032 |
| Parasitic Laelapidae count | 1.017 |
| Myobiidae count | 1.112 |
| Myocoptidae count | 1.080 |
| Listrophoridae count | 1.057 |
| Trombiculidae count | 1.021 |
